# Capture of circulating metastatic cancer cell clusters from lung cancer patients can reveal unique genomic profiles and potential anti-metastatic molecular targets: A proof-of-concept study

**DOI:** 10.1371/journal.pone.0306450

**Published:** 2024-07-31

**Authors:** Kourosh Kouhmareh, Erika Martin, Darren Finlay, Anukriti Bhadada, Hector Hernandez-Vargas, Francisco Downey, Jeffrey K. Allen, Peter Teriete

**Affiliations:** 1 PhenoVista Biosciences, San Diego, CA, United States of America; 2 National Cancer Institute Cancer Center, Sanford Burnham Prebys Medical Discovery Institute, La Jolla, CA, United States of America; 3 TumorGen Inc., San Diego, CA, United States of America; 4 Centre Léon Bérard, Lyon, France; 5 IDEAYA Biosciences, South San Francisco, CA, United States of America; Universite de Nantes, FRANCE

## Abstract

Metastasis remains the leading cause of cancer deaths worldwide and lung cancer, known for its highly metastatic progression, remains among the most lethal of malignancies. Lung cancer metastasis can selectively spread to multiple different organs, however the genetic and molecular drivers for this process are still poorly understood. Understanding the heterogeneous genomic profile of lung cancer metastases is considered key in identifying therapeutic targets that prevent its spread. Research has identified the key source for metastasis being clusters of cells rather than individual cancer cells. These clusters, known as metastatic cancer cell clusters (MCCCs) have been shown to be 100-fold more tumorigenic than individual cancer cells. Unfortunately, access to these primary drivers of metastases remains difficult and has limited our understanding of their molecular and genomic profiles. Strong evidence in the literature suggests that differentially regulated biological pathways in MCCCs can provide new therapeutic drug targets to help combat cancer metastases. In order to expand research into MCCCs and their role in metastasis, we demonstrate a novel, proof of principle technology, to capture MCCCs directly from patients’ whole blood. Our platform can be readily tuned for different solid tumor types by combining a biomimicry-based margination effect coupled with immunoaffinity to isolate MCCCs. Adopting a selective capture approach based on overexpressed CD44 in MCCCs provides a methodology that preferentially isolates them from whole blood. Furthermore, we demonstrate a high capture efficiency of more than 90% when spiking MCCC-like model cell clusters into whole blood. Characterization of the captured MCCCs from lung cancer patients by immunofluorescence staining and genomic analyses, suggests highly differential morphologies and genomic profiles. This study lays the foundation to identify potential drug targets thus unlocking a new area of anti-metastatic therapeutics.

## Introduction

### Significance of metastatic cancer cell clusters

Metastasis remains the primary cause of cancer-related deaths. A recently published clinical analysis identifies metastasis as the cause of death in 50.2% to 90.4% of cases, depending on the origin of the primary tumor [1]. The resulting high metastatic death rates indicate that increased focus on anti-metastatic therapy is urgently needed. Many cancer types have a demonstrated propensity to establish distant metastases among a defined set of organs.

Non-small cell lung cancer (NSCLC), a major malignancy with high mortality, typically metastasizes to bone, followed by brain, liver and adrenal glands [2]. Lung cancer is a prime example of mortality driven by distant metastases to many vital organs [3, 4]. However, the mechanism driving the spread, in particular cancer cell transportation and immune evasion is not well understood. The inability to isolate and profile circulating metastatic cells constitutes a major challenge in beginning to understand these pathways.

Over the past decade, tremendous strides in developing better therapies via molecularly targeted and immune-checkpoint inhibitor treatments, have improved patient outcomes [5]. However, similar significant gains have not been observed for metastatic disease [6]. A key factor in this discrepancy is the complexity of cancer metastasis. Evidence has shown that signaling pathways driving metastatic lesions are often different than found within the primary tumor [7]. A recent publication from Anderson *et al*., representing the Cancer Research UK and Australia Metastasis Working Group stated: “The standard cancer drug discovery and development pathway, including that for molecularly targeted and immunotherapies, generally ignores the ability of experimental medicines to inhibit metastasis.” The authors went on to highlight: “To treat metastasis effectively, we must inhibit fundamental metastatic processes and develop specific preclinical and clinical strategies that do not rely on primary tumor responses.” [8]

Over the last decade, extensive research into individual circulating tumor cells (CTCs), has begun to shed light on tumor proliferation however, it has not produced a unique CTC genomic profile reflecting metastatic pathways across multiple tumor types [9]. Adding further complexity in understanding these mechanisms, the majority of individual cancer cells in circulation undergo anoikis or are eliminated by the immune system [10]. However, an important smaller subset of CTCs circulates in the bloodstream as a cluster of two or more cells. Compelling evidence has demonstrated that CTC clusters have a significantly higher tumorigenic potential once they enter circulation as opposed to individual CTCs [11–13].

Regarding circulating clusters or metastatic clusters, publications have referred to them with different nomenclature such as: circulating tumor cell clusters (CTC clusters), [14] circulating tumor micro-emboli (CTMs), [15] clustered circulating cancer stem cells (cCSCs), [16] metastatic cancer cell clusters, [17] or by using a more general term called collective cell migration, [18] yet all share common characteristics such as a being composed of 2–50 cells with up to 100-fold higher metastatic potential than individual CTCs [14, 19]. Here we will use the terms MCCCs and CTC clusters interchangeably to refer to these circulating, highly metastatic entities.

A number of recent studies have begun to elucidate the phenotypic composition of these MCCCs and have shown them as either homotypic, composed of all cancer cells, or heterotypic, containing multiple cell types including cancerous plus non-cancerous cells [20–22]. The MCCC phenotype possesses an adaptive mechanism that enhances their survival in the harsh bloodstream environment contributing to their metastatic potential [23–26]. Specifically, heterotypic MCCCs demonstrate an increased ability to evade immune surveillance which is postulated to be a result of cancer cell and immune cell communication within the cluster [27–29]. The metastatic cancer cell clustering phenomenon is also thought to mitigate an immune response from natural killer (NK) cells, due to surface receptors, in addition to intercellular communication present within CTC clusters [30, 31].

New drug therapies, focused on MCCCs, will have the potential to dramatically reduce metastasis and improve patient survival. A landmark paper published in 2019 by Aceto *et al*. demonstrated that targeting MCCCs significantly reduces metastasis in an orthotopic breast cancer model in mice [32]. Genomic analysis of breast cancer clusters from patients revealed 10 differentially regulated pathways unique to metastatic clusters [32]. Cardiac glycosides, an existing class of drugs targeting one of those pathways, was evaluated in an *in vivo* animal model. Treatment with one of the drugs (Ouabain) reduced the number of CTC clusters by 60% and decreased metastasis to the lungs and other organs by 80-fold. An intriguing aspect from this work is that the Ouabain dosage levels utilized are not cytotoxic and only disrupted the MCCCs leading to a reduced metastatic index *in vivo*. This study strongly supports the necessity to characterize the MCCCs from patients, to identify previously unseen therapeutic targets and ultimately develop novel drugs thus opening a completely new paradigm in anti-metastatic therapy.

### Current tumor cell isolation methods from whole blood

Insights into how solid tumors propagate to specific pre-metastatic sites is necessary to develop focused therapeutic approaches that can prevent metastasis. While the importance of MCCCs has been well established, technologies to capture and characterize the MCCCs remain problematic and has limited research into this field.

Most existing technologies used to capture CTC clusters or MCCCs, rely on systems designed to capture single CTCs. Many technologies are based on a size filtration mechanism for capture, relying upon the premise that individual CTCs are larger than most leukocytes [33, 34]. Using size filtration, patient blood samples are processed and then sorted based on the individual cell diameter. Whole blood consists of three main components: platelets, red blood cells (RBCs), and white blood cells (WBCs). White blood cells, which are the largest of the three components, are generally 12–15μm in size. Composed of multiple cells, MCCCs are presumed larger than WBCs when present in whole blood, thus would be isolated by size for further profiling. Although the main benefit of size-based technology is that it’s label-free and requires little pre-processing, it has major limitations when it comes to successfully identifying and isolating several types of individual CTCs and MCCCs. It is possible for smaller MCCC’s and many CTC’s to be falsely binned as white blood cells, since the average cell diameters of CTC’s fall within the 10–15μm range [35–37]. A recent review also stated that microfluidic channel size can induce critical flow velocities resulting in shearing and disaggregation of CTC clusters [19, 38]. Another review by Amintas *et al*. concluded that individual CTC isolation techniques are typically not compatible with CTC cluster isolation. They identified three key requirements stating: “Future developments should aim towards methods that allow phenotypic, molecular, and even functional analyses” [39].

### New metastatic cancer cell cluster isolation method

We have developed a highly selective capture technology with a positive capture bias toward MCCCs by specifically exploiting multiple features unique to MCCCs. Their size and thus specific margination behavior during microfluidic flow conditions, [40, 41] as well as overexpression of CD44 on the surface of most cell types found in MCCCs can be utilized for capture from whole blood [42]. The CD44 surface expression is an abundant marker of MCCCs, upregulation of which closely correlates to their metastatic potential [43–47]. Our selective capture approach is based on a biomimetic CD44-induced cell cluster margination effect combined with immobilized capture antibodies coated within the inner walls of a microfluidic chip.

We are reporting on the success of our highly selective capture platform, focused on lung cancer, that preferentially isolates cell clusters predisposed to metastasis. Our technology allows routine and efficient capture of MCCCs providing insights into their biology as well as elucidation of new anti-metastatic therapeutic targets. Ultimately, this will expedite the development of new drugs capable of disrupting clusters and/or cluster formation, thus reducing metastasis.

## Materials and methods

### Microfluidic chip manufacturing and coating procedure

Design, manufacturing, and coating of the microfluidic chips has been published previously [48]. Production of the microfluidic chips used for this study were outsourced to a commercial partner, μFluidix (Scarborough Ontario, Canada). Each chip was produced from two separate layers of polydimethylsiloxane (PDMS) which were then precision aligned to create our open channel microfluidic chips. The design parameters include 32 individual channels (100μm H x 200μm W each) along with a ‘ceiling’ feature composed of alternating patterns of recessed chevrons, 50μm high which induces chaotic flow [48].

Ultrapure pharmaceutical grade alginate (Novamatrix; Sandvika, Norway) used for the hydrogel substrate of the Smart-Coating™, was derivatized with streptavidin (AAT Bioquest, Cat.#16885) molecules using carbodiimide chemistry via the carboxylic groups of the alginate sugar residues [49].Coating of the microfluidic chips with a streptavidin derivatized, calcium (Ca^+2^) cross-linked alginate hydrogel was performed as described previously [48] A secondary coating consisting of a mixture of biotinylated hyaluronic acid and biotinylated monoclonal antibodies (mAbs) was added to the alginate hydrogel. The secondary coating was prepared using cosmetic grade hyaluronic acid (HA) powder (Resurrection Beauty; Cat.# High-MW-1800kDA) that was biotinylated and Cy5-labeled (1:1:1 molar ratios) using carbodiimide chemistry via the carboxylic groups of the glucuronic acid moiety using Biotin-Hydrazide, (Thermo Scientific, Cat.# 21340; and Cy5-Hydrazide from ApexBio, Cat.# A8145). The labeled HA plus a combination of biotinylated monoclonal antibodies was mixed at a 1:16 ratio into phosphate buffered saline (PBS, 1X Caisson Labs PBL01-500ML) along with 0.1% Tween-20. The 4.5 mL mixture was infused onto the alginate coated chip at 200 uL/min and the chip was subsequently washed with 2.5 mL of PBS at 2.5 mL/min. After the second coating was complete chips were stored in a sealed conical tube containing PBS at room temperature until needed. The S1 Fig in S1 File shows a successful coating of our chip with the fluorescently labeled biotinylated hyaluronic acid-Cy5 (bHA-Cy5) bound to the streptavidin derivatized, cross-linked alginate hydrogel. The fluorescent image of S1 Fig in S1 File also shows a captured model MCCC which is a heterotypic spheroid composed of NSCLC A549-GFP (green) co-cultured with human foreskin fibroblast (red, HFFs) cells.

Three surface epitopes, EGFR (ERBB1), MET and HER3 (ERBB3) were selected based on published reports that they are typically expressed on the surface of lung cells [50–52]. Three commercially available antibodies that bind to these epitopes were selected: biotinylated anti-EGFR (Santa Cruz Biotechnology, Cat.# SC-120B) biotinylated anti-MET (Cell Signaling Technology, Cat.#64526BC) and biotinylated anti-HER3 (LS Bio Cat.#LS-C87995). All purchased antibodies were tested to confirm cell surface binding on established NSCLC cell lines, HCC827 (ATCC, CRL-2868) and A549 (Angio-Proteomie cAP-0097GFP) by immunofluorescence (IF). Cell line authentication was based on vendor information provided. See S1 File.

Experiments to assess the microfluidic chip capture efficiency were conducted with multi-cellular heterotypic spheroids, generated using established cell lines co-cultured with the non-cancerous human foreskin fibroblasts, HFF cells, as cancer associated fibroblast surrogates. A known number of model NSCLC MCCCs using co-cultured spheroids (1:1 ratio of NSCLC HCC827 and non-cancerous human foreskin fibroblasts, HFF cells) were spiked into normal donor whole blood samples and processed in triplicate. Results comparing zero antibodies, (Smart-Coating™ biotinylated hyaluronic acid / alginate hydrogel only) to 1 antibody (anti-EGFR) and 3 antibodies, (anti-EGFR, anti-MET and anti-HER3 mAbs combined) were performed. See the S1 File Materials and methods section, along with S2 and S3 Figs in S1 File for additional details on co-cultured spheroid preparation, culture times, staining procedures, and blood processing schematic.

### NSCLC patient whole blood processing

A total of eight patient samples (mean age male [n = 3]: 70.9, ssd 1.1; female [n = 5]: 57.1, ssd 1.2) were collected from the University of California, San Diego (UCSD) Moores Cancer Center, an NCI-Designated Comprehensive Cancer Center. Biospecimens were collected by the Moores Cancer Center Biorepository from consenting patients under a University of California, San Diego Human Research Protections Program Institutional Review Board approved protocol (HRPP# 181755). Biorepository subjects written consent is maintained in the Biorepository archives. Patients were enrolled into the study from July 7^th^ through August 4^th^, 2022. All patients received comprehensive study information from the UCSD biorepository (BR) coordinator and provided written consent as described in section D of the IRB approval form. Patients previously diagnosed with non-small cell lung cancer (NSCLC), at stages III-A, III-B, III-C, IV-A, IV-B agreeing to participate in a study, graciously provided whole blood samples. Non-identifying patient information was collected for each blood sample, including age/gender, confirmed diagnosis, and cancer stage, at the time of blood draw. Patient’s sex and demographic information was not considered as inclusion or exclusion criteria for this study. Future studies involving a higher number of patients will consider these factors to help understanding existing disparities. Sample processing was blinded to patient information.

Collection tubes were treated with EDTA and Tirofiban to prevent coagulation and platelet aggregation respectively [53]. Samples were transported on the same day at ambient temperature and processed through our microfluidic system within 8 hours. In an effort to preserve sample integrity to the fullest extent, no red blood cell (RBC) lysis or whole blood pre-processing was performed, since reports of CTC loses have been noted due to pre-treatment methods [54, 55]. The entirety of the patient collected sample was aspirated into a 10mL syringe, attached to a syringe pump and processed through our coated microfluidic chip at a flow rate of 100μL/minute.

Flow was not disturbed until the entire sample had been infused through the microfluidic chip. After processing our patient blood sample, the chip was flushed with PBS at a rate of 100μL/minute for 5 minutes to remove any remaining unbound blood or white blood cells. A 4% formaldehyde, methanol-free (PFA) solution was then pumped through at 100μL/minute followed by another PBS wash for 5 minutes. Five of the patients’ samples were processed for immunofluorescence imaging and analysis. Cells were stained with a 10 minute flush of our staining palette consisting of Hoechst 33342 live nuclear stain, (Thermo Scientific Cat.# H3570), anti-CD44-FITC (Abeomic Cat.# 10-7516-F) and anti-EGFR-AF594 (Santa Cruz Biotechnology Cat.# SC-120-AF594) all diluted in PBS, followed by a 5 minute PBS wash, at the 100μL/minute flow rate.

Our processed chip was imaged across all flow channels using the Yokogawa CQ1 scanner by stitching together 936 fields captured using a 10x objective. Chips were scanned for Brightfield, Hoechst staining (405nm), CD44 (488nm), EGFR (594nm), and our bHA-Cy5 chip coating (647nm). A relative increase of CD44 and EGFR fluorescent signal against background, including well-defined nuclei morphology, were essential gating parameters used to identify potential MCCCs. Using our 10X stitched composite image as a map for locating potential MCCC candidates, we re-imaged at pre-selected points using a 40x objective to confirm MCCC identification. Using a relatively strict inclusion criteria, any irregular nuclei illuminated via the Hoechst signal even if coupled with positive CD44 and EGFR signal were rejected as MCCC candidates. Furthermore, at least 3 individual nuclei had to be discernable in order to be classified as a candidate MCCC.

### NSCLC whole blood processing for RNA-seq analysis

Three additional patient LC6, LC7, LC8 (selected at random, see Table 1) whole blood samples were processed for RNA-sequencing analysis. Following whole blood infusion, a modified staining protocol was used to reduce imaging time to enhance RNA stability. The chips were flushed with Hoechst 33342 stain (without adding the anti-EGFR and anti-CD44 mAbs), at 100uL/min. for 15 minutes. The Hoechst stain was flushed out using a DMEMs-phenol red media (Thermo Fisher, Cat.# 11965084) at 100uL/min. for 5 minutes‥

**Table 1:** Basic patient information and the number of MCCCs confirmed.

Patient	Age/Gender	Diagnosis	Stage	# Clusters Detected	Detection Method
LC1	82 / M	lung adenocarcinoma	IVb	2	MCCC detection by bright field microscopy and confirmation by immunofluorescence staining for CD44, EGFR, Hoechst
LC2	53 / F	squamous cell lung carcinoma	IV	1	
LC3	59 / F	metastatic lung adenocarcinoma	IV	2	
LC4	65 / M	squamous cell lung carcinoma	IV	1	
LC5	67 / M	non-small cell lung carcinoma	IIIa	3	
LC6	64 / F	lung adenocarcinoma	IV	MCCC were not IF stained to preserve RNA quality	MCCC detection by bright field microscopy, samples processed for genomic analysis by RNA-seq.
LC7	46 / F	non-small cell lung cancer	IV		
LC8	66 / F	non-small cell lung cancer	IV		
Normal Blood Donors	54 / F	N/A	N/A	0	Neg. control
	68 / M			0	

The Inlet and Outlet ports were sealed and imaged using fluorescence microscopy. Brightfield and 405nm images were collected to allow visualization of the captured MCCC nuclei. Post imaging, RNA lysis / stabilization reagent (TaKaRa, Cat. # STO948) was flushed through the microfluidic chip and incubated at zero flow for 15 minutes. Following lysis, fractions were collected at the Outlet port every 15 seconds providing a total of 8 fractions, each with an approximate volume of 25μL. Fractions were immediately frozen at -20°C and sent for RNA sequencing analysis.

One of the patient samples, LC6, was submitted for RNA-seq analysis. This sample corresponded to a 64-year-old female patient, who was diagnosed by transthoracic biopsy with a TTF-1 positive Stage IV lung adenocarcinoma that had metastasized to the brain. Patient LC6 was negative for any EGFR mutations, is a non-smoker, and was on Brigatinib therapy at the time of blood collection. Three RNA lysis aliquots of the LC6 sample were processed and library preparation completed on the 3 aliquots.

### RNA-seq process and bioinformatics analysis

llumina sequencing was performed on RNA extracted from the captured MCCCs. Three RNA aliquots of the LC6 sample were processed for library preparation using the SMART-Seq® HT Kit which combines both cDNA synthesis and amplification (TaKaRa Cat.#634455) plus Nextera® XT Sample Prep Kit (Illumina Cat.#15032354) for tagmentation. Standard Illumina bioinformatics analysis was used to generate fastq files, followed by quality assessment. [MultiQC v1.7 https://multiqc.info/]. The DupRadar plot in the MultiQC report confirmed linear amplification. Fastq files displayed Phred Quality scores ranging from 31.64 to 33.39, along with high alignment (~95%) yet low gene assignment (~5% rates). Low assignment rates were explained by the small amount of MCCC DNA used in library preparation. All downstream bioinformatic analyses were done with R/Bioconductor packages. ‘Rsubread’ v2.10.4 was used for mapping to the hg38 genome and creating a matrix of RNA-Seq counts. Next, a DGElist object was created with the ‘edgeR’ package v3.38.1 [https://doi.org/10.1093/bioinformatics/btp616]. After filtering-out lowly expressed genes, normalization for composition bias was performed using the TMM method (trimmed mean of M-values) as proposed by Robinson and Oshlack [56]. After normalization, genewise exact tests were computed for differences in the means between groups, and differentially expressed genes (DEGs) were extracted based on an FDR-adjusted p value < 0.05.

For RNA-seq integration with datasets GSE74639, GSM18494, and GSM18950, count data corresponding to each of these datasets was downloaded directly from the GEO repository and combined (separately for each dataset) with the common features of the MCCC counts in a single DGElist object. After preprocessing with ’edgeR’, batch correction was performed with the ’removeBatchEffect’ function of the ’limma’ package, using the dataset of origin as the batch variable. Visualization of normalized gene expression data was done with the ’ggplot2’ package.

Datasets used for comparison to the LC6 technical replicates, were the following:

- GSE74639: RNA-Seq of human lung tumor circulating single cells (SCs, aka CTCs) and primary lung tumor cells (PTs) from an orthotopic lung xenograft model [57, 58].
- GSM18949 and GSM18950 are datasets identifying novel genes specific for human lung tissue [59, 60].

The published gene expression datasets were downloaded from the Gene Expression Omnibus (GEO) website. See S1 File for additional bioinformatic analysis details and the specific dataset links. Also, see the S1 Table in the S1 File section for the Key Resources Table which has vendor and catalogue numbers for the key reagents and cell lines utilized for this publication.

## Results and discussion

### Capture optimization

Our strategy was to develop a more ‘selective’ technology within a microfluidic system that would preferentially isolate and capture specific metastatic cell clusters rather than utilize a filtration-based or other size-based methodology. The premise was based on the extreme rarity of MCCCs among billions of red and white blood cells thus promoting the need to use an existing molecular biology process to enhance our cell selectivity. Leukocyte extravasation is a natural process involved with tissue damage, inflammation or infection.[61] Within the circulatory system, binding of CD44-presenting cells to endothelial hyaluronic acid (HA) is the first and essential step initiating margination, rolling, and extravasation [62]. Taking advantage of this existing biological principle, we designed and manufactured a microfluidic chip with a Smart-Coating™ technology that combines a tunable biomimetic and immuno-based dual capture approach [48]. An advantage of using our Smart-Coating™ is the ability to vary the number of monoclonal antibodies and biomimetic tethers which are integrated into the coating of the channel inner walls within the microfluidic chip. Immobilized streptavidin was derivatized on the cross-linked alginate hydrogel as previously described [48]. Antibodies with a biotin conjugate are easily bound to the streptavidin within the coating. Combinations of antibodies as well as biotinylated hyaluronic acid, which serves as a biomimetic tether, make up the second layer of our Smart-Coating. Since our platform is tunable, incorporating different biomimetic tethers and monoclonal antibodies enables the system to be adapted to other solid tumor cancer types.

### Capture antibody selection for NSCLC

**Fig 1:**
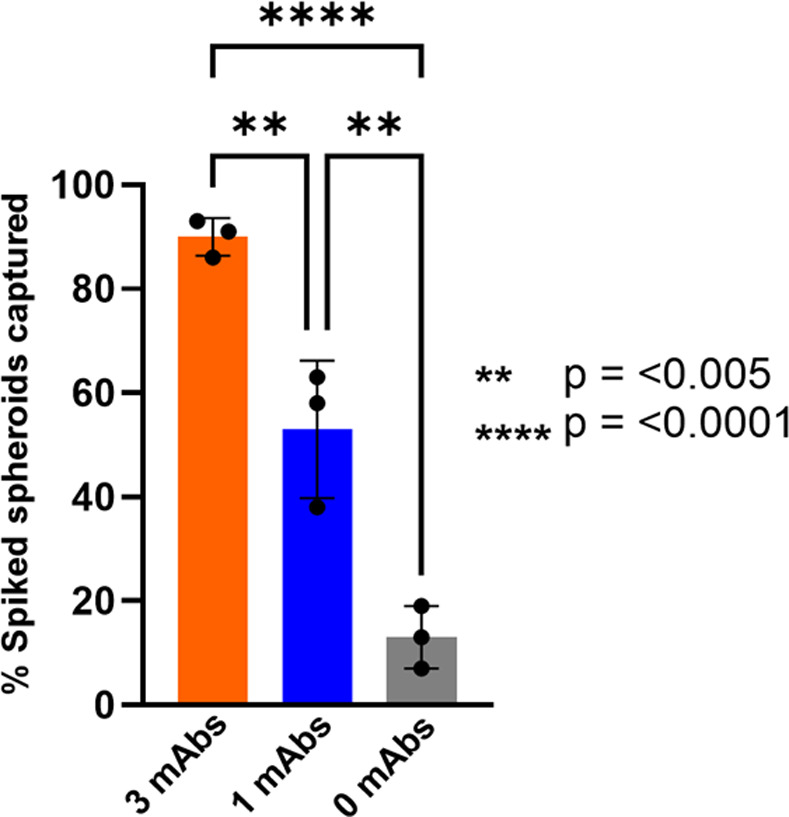
Capture efficiency comparison using varying numbers of tissue specific capture antibodies. The NSCLC co-cultured spheroids were spiked into freshly collected whole blood samples from normal donors. The exact spike count varied for each sample run but fell within the range of 71–91 spheroids. Post processing, NSCLC co-cultured spheroids were quantified via immunofluorescence. Samples were processed in triplicate. One-way ANOVA statistical analysis showed significant differences in capture efficiency between 0, 1, and 3 mAbs (** P< 0.005, *** P<0.0001). 3 mAbs = anti-EGFR, anti-MET, anti-HER3. 1 mAbs = anti-EGFR. 0 mAbs = base Smart-Coating only with zero capture antibodies.

Identifying biomarkers on the surface of lung cells, that are relatively specific for lung tissue, is an important element of our platform. Multiple epitopes were utilized in our immuno-capture method, since this would likely isolate a larger diversity of MCCCs in circulation from different lung cancer patients. Experiments to assess the capture efficiency were conducted using multi-cellular co-cultured spheroids which were counted and spiked into normal whole blood samples. We added close to 100 spheroids per sample (4 mL) to have a sufficient spheroid count to quantify a wide range of capture efficiencies. This approach follows previously described methodologies [63]. The co-cultured spheroids generated using established NSCLC cell lines resemble the MCCCs found in patients’ whole blood. As shown in Fig 1, using 3 capture antibodies resulted in a 90% capture efficiency, thus illustrating an advantage using multiple over a single capture antibody. Since utilizing a higher capture efficiency methodology will likely increase the probability of MCCC capture, 3 capture antibodies were used for subsequent NSCLC patient samples. The ability to capture co-cultured spheroids using the Smart-Coating alone, without any immobilized antibody, is likely due to the CD44 expression found on the co-cultured spheroids that have an avidity for the hyaluronic acid in our second coating [64, 65].

### Patient samples

A total of 8 patient samples were collected from the UCSD Moores Cancer Center and processed through our Smart-Coated™ microfluidic chips that incorporated the 3 mAbs which demonstrated the highest capture efficiency. Two additional normal, healthy donor, patient whole blood samples were processed from a 54-year-old female and 68-year-old male, both of which revealed no confirmed MCCCs.

**Fig 2:**
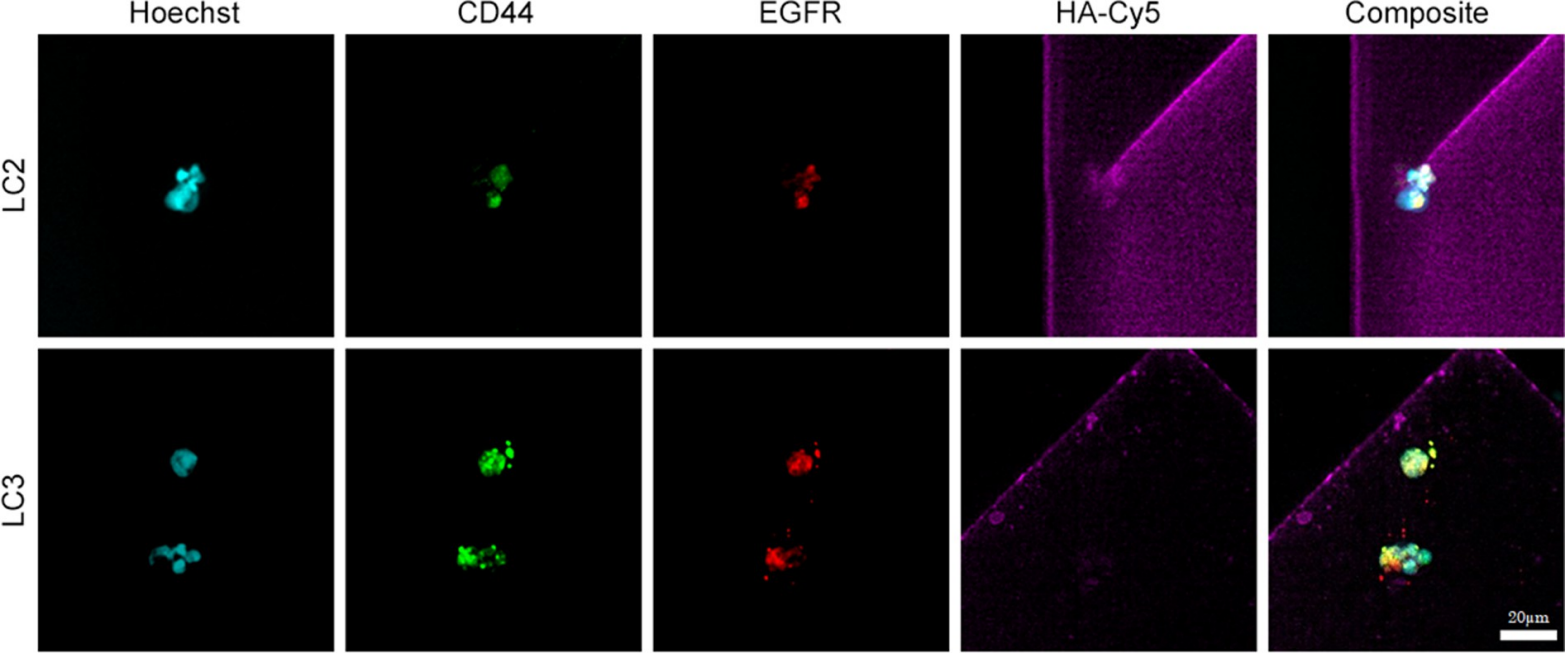
Representative 40X objective IF images of microfluidic chip captured NSCLC MCCCs from the whole blood of two NSCLC patients. Nuclei were stained with Hoechst 33342 (405nm blue) plus surface antigens with anti-CD44-FITC (488nm green) and anti-EGFR-AF594 (594nm red). The Smart-Coating™ is visualized via HA-Cy5 (647nm purple). The overlapping of both CD44 and EGFR fluorescence (yellow) in the composite image indicates cells expressing both markers. Two separate MCCCs were observed for patient LC3. Scale bar = 20μm for both LC2 and LC3 images.

Five of the patient samples, following processing on the chip, included immuno-fluorescence imaging for confirmation of the captured MCCCs using conjugated monoclonal antibodies as listed in Fig 2.

Detailed fluorescent image analysis of all microfluidic channels processed for each patient revealed MCCCs were identified and confirmed in all 5 samples. Basic patient information including diagnosis and disease staging plus detection method are listed along with the number of confirmed MCCCs, each consisting of a minimum of 3 morphologically distinct nuclei (Table 1).

The rationale for the selected surface antigens to confirm MCCCs is that the overexpression of both CD44 and EGFR is associated with cancerous lung cells [16, 43, 66]. Elevated fluorescent signal of anti-CD44 mAb conjugate and anti-EGFR(528) mAb combined with at least 3 distinct nuclei with well-defined nuclear morphology (Hoechst 33342) were essential gating parameters used to identify MCCCs. Observations of the captured MCCCs appear to show clusters with multiple nuclei that were relatively compact and not significantly larger than some single cell, non-cancerous monocytes. See Fig 2 [67, 68]. The observation of platelets associated with MCCCs, as reported in the literature, was not expected in our captured clusters since pretreatment with tirofiban disrupts platelet aggregation. Tirofiban treatment has been shown to increase CTC and MCCC surface antigen accessibility [53, 69, 70]. The remaining three NSCLC patient samples were processed with a modified protocol without immunofluorescence to help ensure RNA stability was maintained for subsequent ultra-low concentration bulk RNA sequencing analysis.

Imaged cell clusters were binned into three groups:

1. Hoechst (+), EGFR (-) and CD44 (-) Disregarded
2. Hoechst (+), CD44 (+) and EGFR (-) Disregarded
3. Hoechst (+), CD44 (+) and EGFR (+) Confirmed MCCC

Cells positive for only Hoechst and CD44 were presumed to be white bloods cells [71]. Any clusters with green or red positive staining without clear nuclear morphology via Hoechst staining were disregarded as potential MCCCs. Only clusters with all three stains, including 3 well defined nuclei were considered MCCCs. Examples of the confirmed NSCLC MCCCs captured from whole blood are shown in Fig 2. Note the heterogeneous shapes and sizes plus the multi-cellular composition as seen by the variation in fluorescence signal distribution among the cells in the MCCCs. A single MCCC was observed in patient LC2 which appears to have 5 nuclei. Also note the apparent compact cellular morphology of the clusters. Two separate, multi-cellular MCCCs were observed in patient LC3. The upper LC3 MCCC appears to have 3 separate nuclei and the lower MCCC has 4 nuclei present.

### MCCC characterization via RNA-seq analysis

Determining the unique gene expression profiles represented within the MCCCs via RNA-seq analysis is crucial to begin learning more about the mechanisms of metastasis. Specifically, the ability to characterize the metastatic cancer cell clusters, captured while in transit, directly from a whole blood specimen will begin to shed light on the numerous signaling pathways involved in the metastatic cascade. Understanding the unique gene expression profile emanating from MCCCs found in NSCLC patient’s whole blood is essential in discovering new anti-metastatic targets for therapeutic development. Previous research has reported MCCCs associated with fibroblasts, neutrophils, macrophages and platelets [70, 72, 73]. As mentioned above, heterotypic MCCCs have an immune survival advantage, thus all cell types should be included for sequencing to elucidate a complete profile [27–29]. The RNA-seq analysis produced from the captured MCCCs represents a profile from all captured cells, including both cancerous and non-cancerous cell types. Evidence of non-cancerous cells being captured was noted in the low-level expression of *PTPRC* (aka *CD45*), *CD163*, *CD99*, and *LYZ* which are common on monocytes and other WBCs (See, S1 Data) [74–76]. However, our use of a selective capture methodology including rigorous washing minimizes the number of non-specifically bound cells that are unrelated to the MCCCs. Visualization via fluorescence microscopy of all microfluidic channels confirmed few other extraneous cells were present. [Data not shown]

Three additional patient samples with confirmed NSCLC diagnoses were processed through our microfluidic chips, using an abbreviated protocol, for the purpose of identifying gene expression profiles represented by the MCCCs. Following a modified processing method, MCCC capture was confirmed with Hoechst staining of the clustered nuclei. After imaging, the microfluidic chip was infused with RNA lysis/stabilization buffer, incubated and then aliquoted fractions were collected at the outlet port.

RNA-seq analysis was performed on the MCCCs captured from the 6^th^ patient, designated LC6, who is a 64-year-old female with TTF-1 positive Stage IV lung adenocarcinoma. A 10mL whole blood sample was processed using our Smart-Coating™ microfluidic system. Post analysis imaging revealed multi-nucleated cell clusters (each with 3 or more nuclei based on Hoechst staining). RNA-seq analysis was performed on 3 separate lysate aliquots eluted from the chip processed from patient LC6. MCCC RNA sequencing data was of good quality, with high alignment rates to the human hg38 assembly (above 85% for the 3 technical replicates), Only a small fraction of reads (~ 5%), were assigned to genes, likely due to the low number of MCCC cells in the sample (S1 Data). Despite such a low assignment rate, approximately 7000 genes displayed at least 10 counts in the 3 MCCC samples (S2 Data). Moreover, 138 genes were highly expressed (over 1k reads) consistently across the 3 replicates (S3 Data).

**Fig 3:**
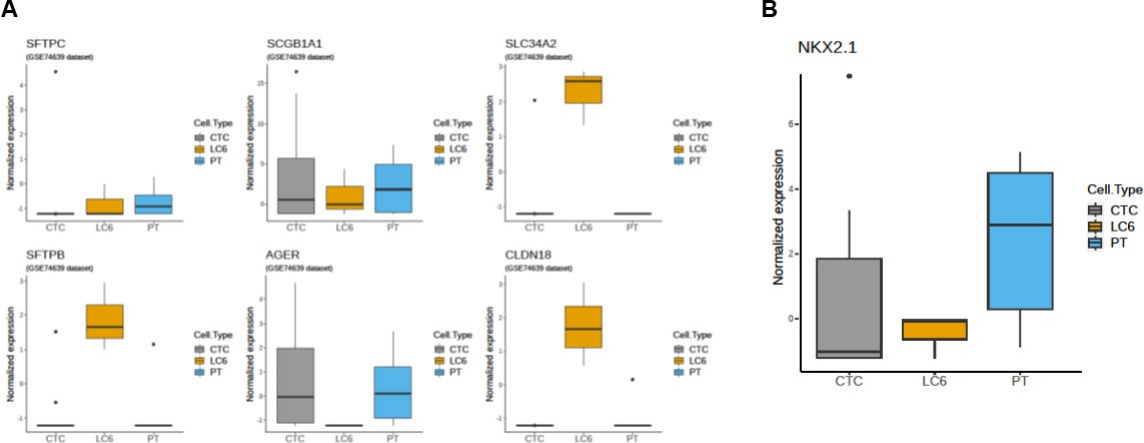
Normalized RNA-seq gene expression on a logarithmic y axis of 6 published genetic markers correlated to human lung tissue. **(A)** Plots shown are the comparisons of the MCCCs (patient LC6) to lung individual cells (CTCs) and lung primary tumor cells (PT) for those selected markers. The plots from 5 of the 6 markers selected confirm the lung cell origin of the MCCCs. **(B)** Boxplot showing the normalized gene expression of *NKX2*.*1* (aka *TTF-1*) of the MCCCs compared to both lung CTCs and lung primary tumor samples (PT). The detection of *TTF-1* from lung biopsy specimens is typically used to aid in the diagnosis of NSCLC.

Bioinformatic analysis of the RNA-seq data was used to confirm that the MCCCs captured originated from lung tissue. Expression levels were based on 6 well known genes found in lung cells from previously published datasets of human lung tissue, GSM18949 and GSM18950 [59, 77, 78]. The boxplots shown in Fig 3 also included normalized gene expression levels found in lung CTCs and lung primary tumor cells published elsewhere. This RNA-seq dataset (GSE74639) is comprised of lung tumor circulating single cells, designated (SCs) in the original manuscript, but also referred to here as CTCs as well as lung primary tumor cells (PTs) [57, 79].

Expression of five out of the six genes assessed were found within the MCCCs from patient LC6: *SFTPC*, *SCGB1A1*, *SLC34A2*, *SFTPB* and *CLDN18* thus confirming the tissue of origin for the captured MCCCs was lung. Another gene was identified within the captured MCCCs, *TTF-1* (aka *NKX2*.*1*), which is commonly used to aid in the diagnosis of primary pulmonary adenocarcinoma. The *NKX2*.*1* expression is found in 70–90% of non-mucinous adenocarcinoma subtypes [80]. The boxplot shown in Fig 3 reveals the expression of *TTF-1*(*NKX2*.*1*) in the MCCCs from patient LC6, which is consistent with the patient diagnosis obtained from the UCSD Moores Cancer Center, see Table 1. Data supports the finding that the MCCCs are from lung tissue and are adenocarcinoma cells.

**Fig 4:**
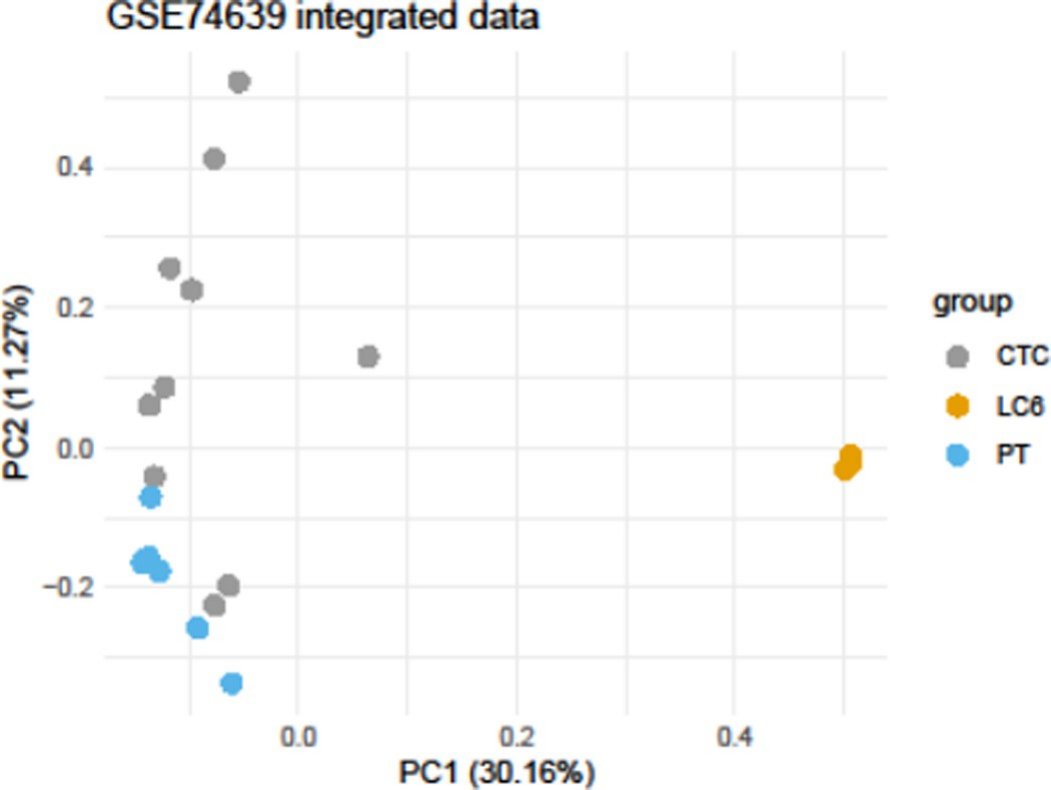
Principal Component Analysis (PCA) of the integrated data sets. The plot compares lung circulating tumor cells (CTC), lung primary tumor cells (PT) and the 3 MCCC aliquoted samples (LC6). The PCA plot of the patient’s MCCCs sequence data are grouped separately compared to the other lung cancer cell types.

Unfortunately, no RNA-seq data was available from our patients’ primary tumor. Also, no datasets on aggregates or CTC clusters (aka MCCCs) from lung cancer patients are publicly available. Therefore, we utilized publicly available gene expression datasets from different NSCLC patients in comparison to our MCCC patient expression data. Using the described NSCLC RNA-seq datasets on primary tumor cells (PT) and individual CTCs (see Materials and methods) we performed an unsupervised clustering analysis comparing MCCCs with this data. Shown in Fig 4 is a Principal Component Analysis (PCA) of the integrated data sets comparing lung CTCs, lung PTs, and the 3 MCCC aliquoted samples (LC6). The PCA plot shows a very tight grouping of the LC6 aliquots, as well as discrete but more closely distributed grouping for the CTC and PT data from multiple patients.

**Fig 5:**
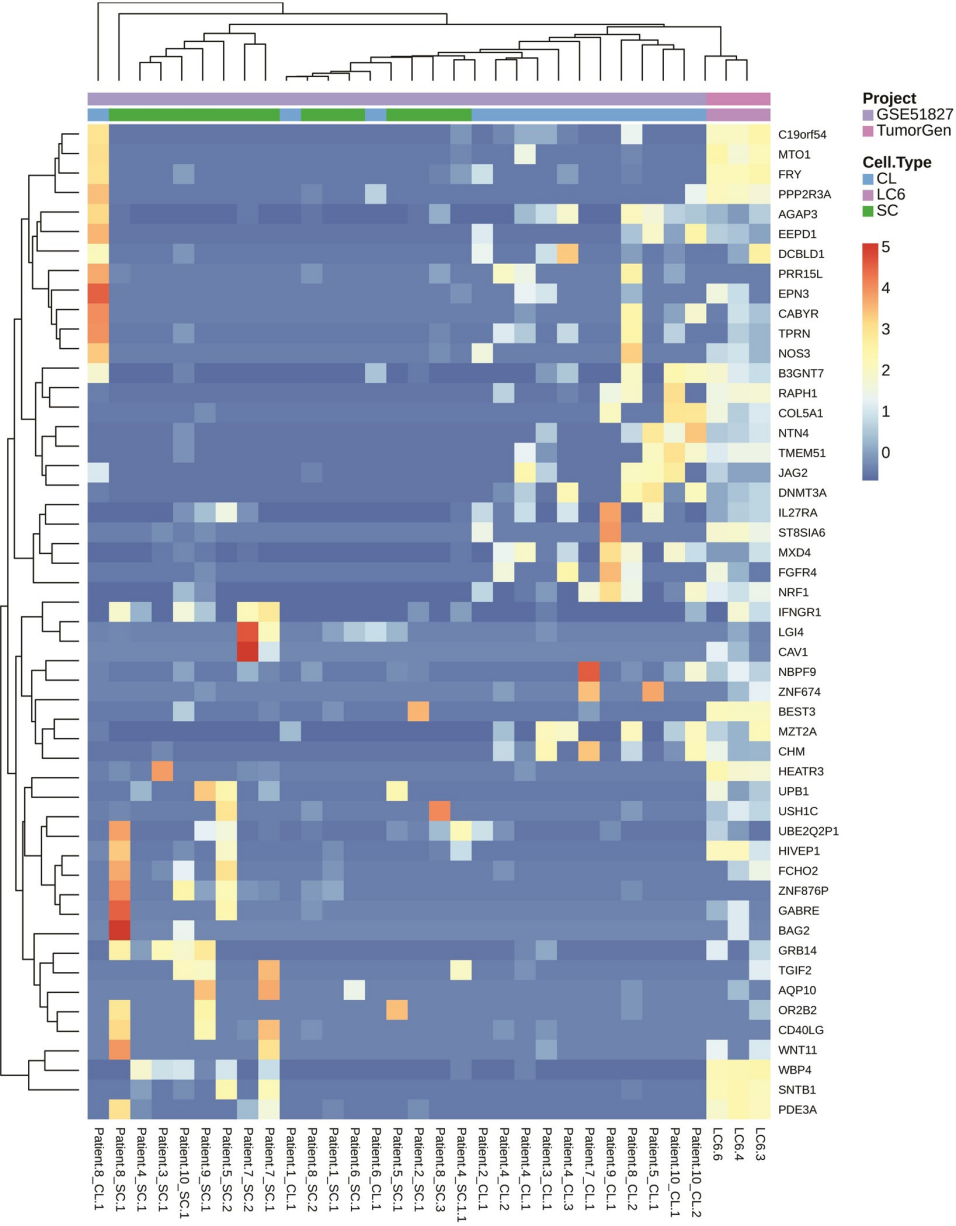
An RNA-seq heatmap generated using differentially expressed gene (DEGs) between PTs, CTCs and MCCCs. Although the MCCCs share some similarities between lung primary tumor cells (PTs) and individual lung circulating tumor cells (CTCs) from different patient studies, the DEG grouping of the MCCCs displays a unique profile.

In order to better visualize a unique gene expression profile, based on differentially expressed genes (DEGs) for the MCCCs, a heatmap was generated showing lung MCCCs relative to individual lung CTCs and lung primary tumor cells. The series GSE74639 RNA-seq dataset was utilized which contained 10 individually sequenced lung CTCs and 6 patient’s lung primary tumor cells which were compared to the 3 aliquots of MCCCs from patient LC6 [57]. The MCCCs from patient LC6 display a discrete expression profile verses lung CTCs and PTs. The heatmap in Fig 5 shows that the MCCC profile shares some common gene expression among all 3 groups but also displays significant differences.

Even though a single MCCC data set is not ideal representing a unique expression profile, the published RNA-seq data sets from multiple patients in lung CTCs and primary tumor cells, segregated by cell type (CTCs vs. PTs), showed highly consistent profiles within a type among each patient. The clustering analysis of the single patient sample in our study is suggestive of a unique gene expression signature MCCCs harbor rather than individual patient differences. We believe that this reflects the contributions from the individual patient samples but also shows the fundamental differences between the origin of the cells sampled. Validation of this uniqueness will require extended patient sample testing incorporating autologous datasets rather than comparisons to published CTC and primary tumor data. However, our proof-of-concept study clearly shows the ability to collect the necessary data to fully characterize MCCC RNA-seq profiles using our approach.

**Fig 6:**
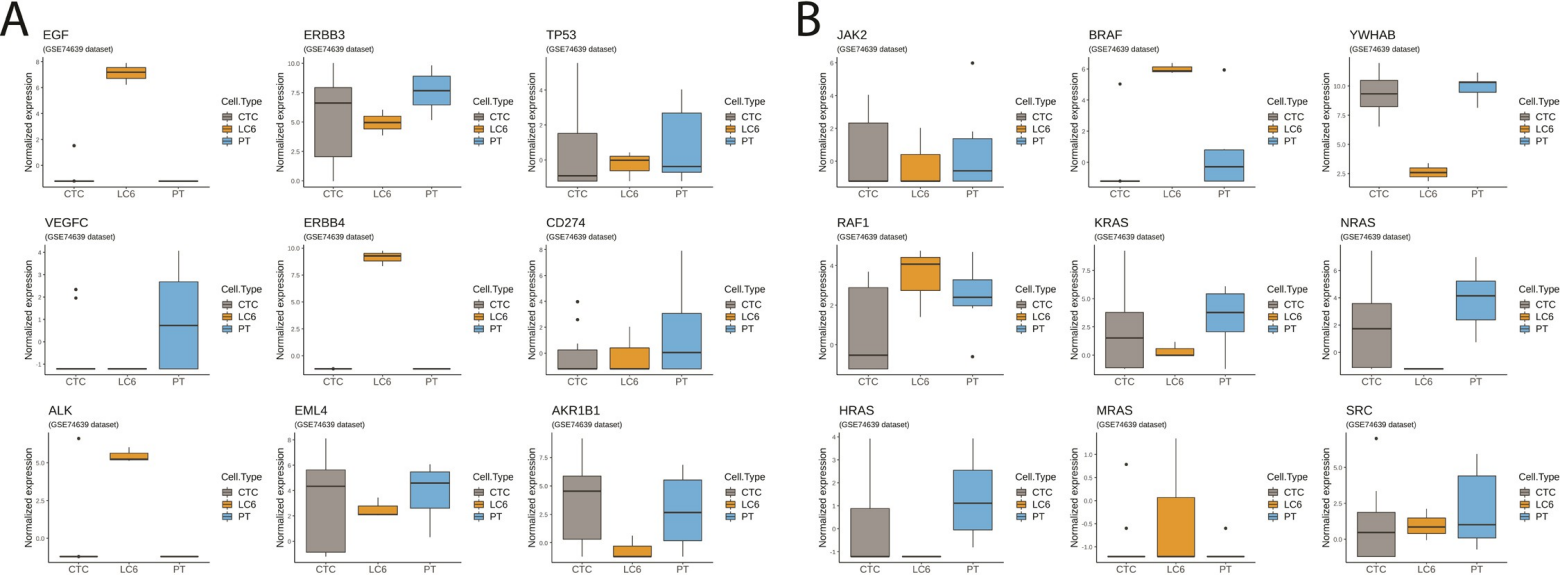
Sampling of potential, actionable therapeutic gene expression targets. Box plots comparing normalized gene expression levels on a logarithmic y axis from a sampling of genes among the MCCCs from patient LC6. **(A)** The selected actionable genes may be targets since existing drug interventions or new therapeutics are in development for those selected. Note the increased expression of *EGF*, *ERBB4* (*HER4*) and *ALK* compared to lung CTCs and lung primary tumor cells. **(B)** An Illustration of several genes within the *Ras/Raf* pathway. Note the relatively high expression of *BRAF* among the MCCCs suggesting a potential unseen therapeutic target not usually observed in lung primary tumor cells.

Fig 6A shows some examples of potentially actionable molecular targets expressed in the MCCC RNA-seq data. Cross-referencing of these targets with the Drug-Gene Interaction Data Base (DGIdb.org), [81] we identified numerous drugs and drug candidates with high interaction scores (S3 Table in S1 File). Some examples show elevated expression in contrast to CTCs and PTs in this dataset (e.g. *ALK*, *ERBB4*, and *EGF*) while other molecular targets while clearly expressed are not noticeably upregulated. S3 Table in S1 File shows a list of corresponding therapeutics available or in development for these molecular targets. Additional research is necessary to demonstrate that inhibiting the identified targets will interfere with metastatic cluster biology.

The RNA-seq analyses from our captured MCCCs have shown the expression of several genes that are considered actionable relative to targeted therapies in the treatment of cancer. An example of a potential, previously unseen therapeutic target among the MCCCs is suggested by the elevated *ERBB4* expression as seen in Fig 6A. The ERBB4 is a tyrosine kinase receptor and a member of the epidermal growth factor receptor (EGFR) family, also known as HER4. The ERBB4 receptor is activated via ligand binding which subsequently impacts MAPKs and PI3K/AKT pathways [82]. Much research continues to elucidate the role of *ERBB4* mutations and expression among many cancer types but it may be associated with proliferative and migratory abilities of NSCLC [83]. While, *ERBB4* mutations and/or high expression levels observed among NSCLC patients have been identified, their clinical significance remains unclear [84, 85]. Elevated levels of ERBB4 among MCCCs, which can be confirmed when additional patient samples are processed, suggests a possible role in the metastatic cascade thus warranting further study.

Another gene with high expression levels within the MCCCs relative to lung CTCs and primary tumor is *BRAF* (See Fig 6B). Detection of mutated *BRAF* has been low and reported in only 2–7% of advanced NSCLC patients [86, 87]. The *BRAF* gene is part of the MAPK/ERK signaling pathway, which plays a crucial role in regulating cell growth, differentiation, and survival. Mutated *BRAF* is a well-known driver of various cancers but not usually observed with lung cancer [88]. Yet, a recent publication identified elevated wild type (WT) *BRAF* expression levels at all stages (I-IV) from lung adenocarcinoma patients and was significantly associated with decreased overall survival [88, 89]. Identifying elevated *BRAF* expression among these rare MCCCs reinforces the need to characterize the metastatic clusters to reveal previously unseen therapeutic targets. However, the possibility that the high *BRAF* expression could have been inherited from the primary tumor and is not a unique profile of MCCCs cannot be disregarded since we do not have corresponding RNA-seq data for this patient. Furthermore, our current data is limited to expression levels and does not inform on mutational status.

An important strategy for disrupting MCCCs (aka CTC clusters) was described by Nicola Aceto’s group where they identified molecules which interfered with intercellular adhesion [32]. Results from an animal model study using patient derived xenografts demonstrated an 80-fold reduction in metastatic index with the adhesion molecule inhibitor treatment group. Following this strategy, we reviewed the MCCC RNA-seq data to identify elevated expression levels for adhesion and epithelial to mesenchymal (EMT) molecules which may be associated with preparing metastatic clusters for intravasation.

**Fig 7:**
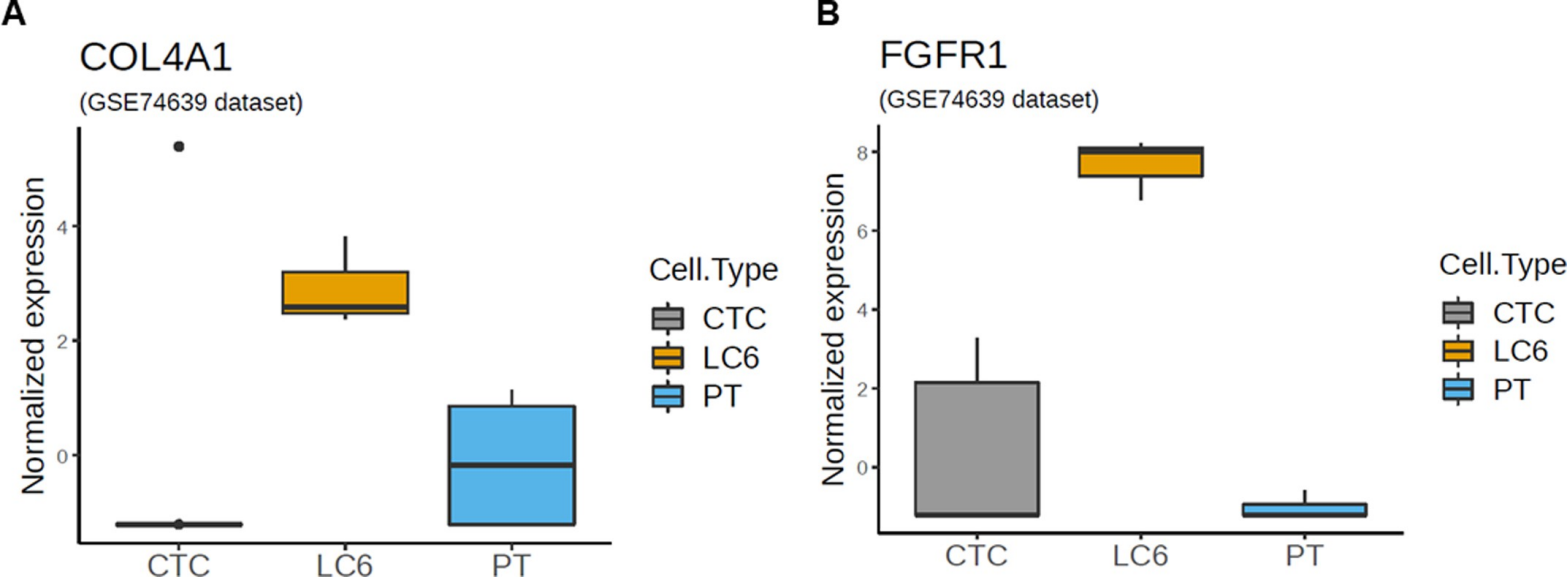
Gene expression correlated to intercellular adhesion and EMT. MCCCs from patient LC6 appear to show relatively high expression levels of *COL4A1* and *FGFR1* compared to individual circulating lung CTCs and lung primary tumor cells.

Two genes associated with adhesion and EMT, *COL4A1* (Collagen, type IV, alpha 1) and *FGFR1 (*Fibroblast growth factor receptor 1), showed elevated gene expression (see Fig 7). The *COL4A1* codes for a component of type IV collagen which comprises a complex protein network involved with basement membranes supporting cell structures [90]. COL4A1 is known to promote the growth and metastasis of hepatocellular carcinoma cells [91]. Collagen, a major component of the extracellular matrix (ECM), plays a significant role in cancer metastasis. Cancer cells can alter the composition and organization of the ECM within the tumor microenvironment. Tumor cells often produce enzymes such as matrix metalloproteinases (MMPs) that degrade and remodel the ECM, allowing cancer cells to invade surrounding tissues more easily [92]. A recent publication discusses targeting specific focal adhesion and ECM receptor pathways associated with COL4A1 for liver and lung metastases. [93].

Also shown is the upregulation of *FGFR1* which has been associated with the lung cancer progression pathway in some studies [94, 95]. FGFR1 is a receptor tyrosine kinase also linked to the *MAPK/ERK* pathway and overexpression of this gene was found to promote epithelial–mesenchymal transition (EMT) plus metastasis‥ Specifically, it is linked to resistance and decreased efficacy of EGFR tyrosine kinase inhibitor (TKI) treatments [94, 96].

Understanding the molecular signaling pathways of metastasis is essential for developing new therapeutics designed to prevent cancer’s spread. Isolating and characterizing the cancer cell clusters which are more tumorigenic versus individual cancer cells is an important precursor for new anti-metastatic drug development [32, 97]. Facilitating the isolation and characterization of MCCCs will increase the number of patients analyzed for these metastatic clusters in real time, as well as during various stages of their disease. Technology that allows for frequent sampling at multiple time points may improve our understanding of the metastatic cascade during cancer’s progression. Additionally, easier access to MCCCs will increase research focus on the mechanisms responsible for cancer cluster intravasation and ultimately aid the development of therapeutics to stop their genesis.

Our proof-of-concept study utilized a completely novel dual capture approach based on a highly selective, biomimetic-immunocapture ‘Smart Coating’ that fast tracks isolation and characterization of extremely rare MCCCs. The Smart Coating™ technology provides a positive selection bias for capturing extremely rare MCCCs directly from unprocessed whole blood.

The RNA-seq heatmap, which suggests a unique DEG profile, (Fig 5) further supports our proof-of-concept study, demonstrating the ability to capture MCCCs in circulation and characterize them to identify potential therapeutic targets. Also, identifying a unique DEG profile for MCCCs will lead to illuminating different signaling pathways that are in effect during the metastatic cascade [9, 98].

## Conclusion

We acknowledge that our study has limitations. Specifically, the number of patient samples processed using fluorescently labeled mAbs to characterize the MCCCs and the corresponding RNA-seq analyses was relatively low. However, the results demonstrating that one or more MCCCs were found in all patient samples processed are highly promising. Also, captured clusters were amenable to immunofluorescent characterization and yielded high quality RNA for detailed genomic profiling. Future studies will further characterize these metastatic vectors using epigenomic and proteomic characterization. Our results show the utility of our dual capture technology to reveal previously unseen metastatic pathways. For example, identification of *ERBB4*, *BRAF*, *COL4A1* and *FGFR1* as molecular targets in highly tumorigenic MCCCs, may serve as focal points for new anti-metastatic drug development. Although the gene expression data is based on replicates from a single patient sample, our successful proof-of-concept study will enable us to expand into higher sample numbers, allowing identification of definitive expression profiles associated with NSCLC MCCCs, shared among many different patients. This will include comparisons of RNA-seq data from autologous patient samples such as lung primary tumor cells and plural effusion derived cells versus MCCCs. The simplicity and utility of our new platform has the potential to greatly expand the study of metastatic cancer cell clusters for many different solid tumor cancers.

## Supporting information

S1 File (DOCX)

S2 File (PNG)

S1 Data (XLSX)

S2 Data (XLSX)

S3 Data (XLSX)

S4 Data (XLSX)
