# Correction: Capture of circulating metastatic cancer cell clusters from lung cancer patients can reveal unique genomic profiles and potential anti-metastatic molecular targets: A proof-of-concept study

**DOI:** 10.1371/journal.pone.0321150

**Published:** 2025-03-25

**Authors:** The PLOS One Staff

**Notice of republication**

This article was republished on 28^th^ February 2025, to correct the publication of an incorrect Figure 5 that was introduced during the typesetting process. The publisher apologizes for the errors. Please download this article again to view the correct version. The originally published, uncorrected article and the republished, corrected articles are provided here for reference.

## Supporting information

S1 File Originally published, uncorrected article. (PDF)

S2 File Republished, corrected article. (PDF)
