# Supplementary material for: Capture of circulating metastatic cancer cell clusters from lung cancer patients can reveal unique genomic profiles and potential anti-metastatic molecular targets: A proof-of-concept study
Source: PLoS One. 2024 Jul 31;19(7):e0306450. doi: 10.1371/journal.pone.0306450 (PMC11290651; doi:10.1371/journal.pone.0306450)
Supplement: S1 File — (DOCX) [file pone.0306450.s001.docx]

# **Title:**

# **Capture of circulating metastatic cancer cell clusters from lung cancer patients can reveal unique genomic profiles and potential anti-metastatic molecular targets: A proof-of-concept study.**

Kourosh Kouhmareh^1,#a^, Erika Martin^1^, Darren Finlay^2^, Anukriti Bhadada^3,#b^, Hector Hernandez-Vargas^4^, Francisco Downey^3,#c^, Jeffrey K. Allen^3^ and Peter Teriete^3,5*^

^1^ PhenoVista Biosciences, 6195 Cornerstone Ct E STE 114, San Diego, CA 92121, United States of America

^2^ National Cancer Institute Cancer Center, Sanford Burnham Prebys Medical Discovery Institute, 10901 N. Torrey Pines Rd., La Jolla, CA 92037, United States of America

^3^ TumorGen Inc., 6197 Cornerstone Ct E STE #101, San Diego, CA 92121, United States of America

^4^ Centre Léon Bérard, 28 Prom. Léa et Napoléon Bullukian, 69008 Lyon, France

^5^ IDEAYA Biosciences, 7000 Shoreline Ct STE #350, South San Francisco, CA 94080, United States of America

^#a^ Current Address: Pathology Department, University of California San Diego, 9500 Gilman Dr., La Jolla, CA 92093, United States of America

^#b^ Current Address: NanoCellect Biomedical, Inc., 9525 Towne Centre Dr. #150, San Diego, CA 92121, United States of America

^#c^ Current Address: Department of Bioengineering, University of California San Diego, 9500 Gilman Dr., La Jolla, CA 92093, United States of America

*Corresponding author

E-mail: pteriete@tumorgen.net

**Supporting Information**

Materials and methods

Our microfluidic system can facilitate the capture of extremely rare metastatic cancer cell clusters (MCCCs). It is the first platform specifically designed for this purpose and includes key features, such as channel dimensions suitable for clusters of varying size and structural features that induce non-homogeneous flow to facilitate immuno-based capture. We further addressed rare cell cluster isolation challenges by developing a dual capture mechanism, which combines biomimicry with immuno-capture. Our dual-capture approach is based on a biomimetic cell margination effect driven by CD44 combined with an immobilized antibody capture molecule. The CD44 surface antigen is an abundant marker of MCCCs, upregulation of which closely correlates to their metastatic potential.[1-5] During the normal biological process of leukocyte extravasation into sites of infection or inflammation, the binding of leukocyte presenting CD44 to endothelial hyaluronic acid (HA), its principal ligand, is the first and essential step initiating margination, rolling, and extravasation.[6-8] Tethering of the CD44^+^ cells by HA allows subsequent interactions, such as PSGL-1 binding to E- or P-selectins as well as VCAM1,[6, 9] to provide full adhesion before diapedesis occurs. The CD44 is further involved in activating signaling pathways required to complete various steps along the active extravasation of the leukocytes.[6, 10, 11] High-jacking of CD44-driven active extravasation further postulates how CD44^+^ MCCCs increase their metastatic abilities. This means selection of MCCCs based on a CD44^+^ phenotype confers a bias of the platform toward malignancy, a concept that is supported in a number of recent studies.[4, 12, 13]

The microfluidic chips received from µFluidix underwent a detailed quality control check before coating. A stitched composite of over 900 brightfield images taken with a 10x objective was scanned and compiled for each chip which was then assessed for structural damage and defects.


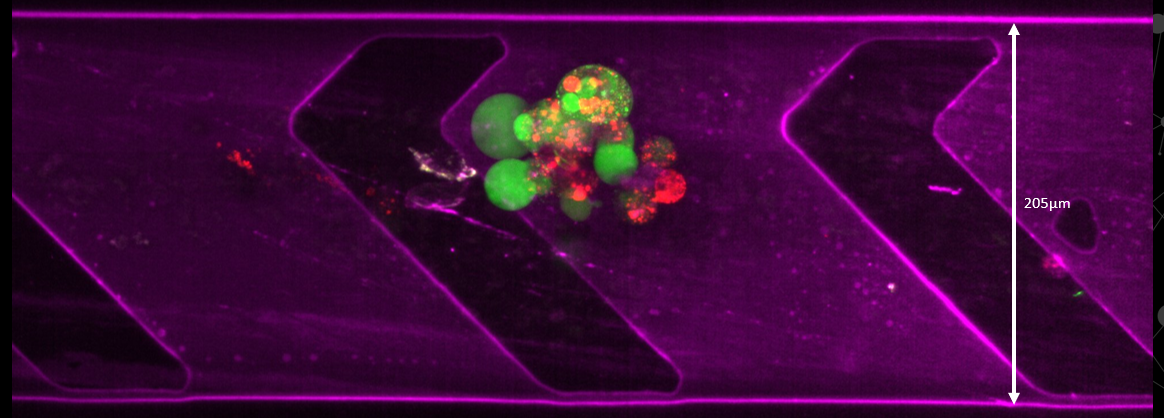


**S1 Fig. Immunofluorescence image of the Smart Coating and a captured model MCCC.** Image of a captured co-cultured spheroid NSCLC A549-GFP (green 488nm) with HFFs (red 568nm) that was spiked into 3 mLs of whole blood and processed through our microfluidic chip. The channel walls shown in (purple 647nm) confirm the presence of our biotinylated HA-Cy5 labeled Smart Coating. Maximum intensity projection (MIP) image was acquired using a 40x objective, on a confocal microscope.

Stability testing of our coating has shown functionality is maintained for more than 12 months after coating when stored in aqueous buffer. Derivatization of hyaluronic acid (HA) (Cosmetic grade, Resurrection Beauty) to incorporate biotin (EZ-Link Hydrazide-LC-biotin, Thermo Scientific, Cat.#21340) functional groups and a Cy5 fluorophore (Cy5-Hydrazide, ApexBio Cat.#A8145) was performed utilizing the same carbodiimide chemistry mentioned earlier.[14].

The capture antibodies used to coat our microfluidic chips were validated to confirm binding to cell surface epitopes using immunofluorescence in both 2D and 3D culture models on our candidate immortalized NSCLC cell lines. All immunofluorescent staining was done on live cells/spheroids to ensure no variations in antibody expression amongst live cells when followed by fixation. Cells were seeded in 384-well low volume (Greiner UV-Star 384-well Cell Culture Treated, COC Microplate Cat.788890) and 384-well Sbio U-bottom plates (Sbio MS-9384UZ). The co-cultured spheroids were incubated for 72 hours. An antibody mixture containing a 1:100 dilution of anti-EGFR, anti-MET, and anti-HER3 respectively in sterile filtered Goat Blocking reagent (PhenoVista Biosciences proprietary reagent) was added and allowed to incubate in-well for 30 minutes. Cells were then fixed with 4% formaldehyde methanol-free (PFA, Cell Signaling Technology, Cat. # 47746P) for 20 minutes and a corresponding Alexa Fluor secondary antibody was added and allowed to incubate for 30 minutes. (Fixation was necessary to preserve the cells due to lengthy microscope imaging times using a 10X objective to collect over 900 images at 4 wavelengths plus bright field, covering all 32 channels in our microfluidic chip).

Cells were imaged using a fluorescent microscope (Yokogawa CQ1 scanner) and analyzed for positive extracellular specific staining. Both 384 microwell plates and our microfluidic chips were imaged utilizing the Yokogawa system. Any cells bound to the chip through our immuno-based capture method remain tightly bound since an elevated flow rate of 200uL/min did not remove any bound MCCCs.

A simplified schematic of the blood sample preparation and microfluidic infusion process is shown in S2 Fig. Experiments were done using a 3D immortalized NSCLC cell line model system, that were spiked into normal patient whole blood to optimize our Smart-Coating platform. A 1:1 ratio of co-cultured spheroids was generated by seeding NSCLC A549-GFP (Angio-Proteomie, Cat. #: cAP-0097GFP) and/or NSCLC HCC827 (ATCC, CRL-2868) cells respectively, in combination with human foreskin fibroblasts (HFF-1) (ATCC, SCRC-1041) into an 384 well U-bottom, clear, ultra-low attachment plate (Sbio, MS-9384UZ). Cells were incubated at 37°C for 72 hours, resulting in compact, tightly formed, ~30 cell spheroid model MCCCs which remained intact during microfluidic processing at 100µl/min. Prior to infusion into the microfluidic chip, HCC827 co-cultured spheroids were incubated in-well with CellTracker Green (Invitrogen cat\#\ C2925) for 1 hour until they were ready to be hand-picked and counted. The exact spike count varied for each sample run but consistently fell within the range of 71-91 spheroids added into a 4mL aliquot of freshly collected whole blood. The freshly collected sample from the San Diego Blood Bank was collected, transported and processed on our microfluidic system within 8 hours of donation and kept at ambient temperature. Collection tubes (BD Vacutainer® tube 10mL K2 EDTA, Cat. # BD-367863) were treated with 10.8mg dry coated K2-EDTA and Tirofiban (SelleckChem Cat.# S8594). Injection of either 120 - 200µL of a 5.0mg/mL Tirofiban stock solution in DMSO, 100ug/mL final concentration, was transferred into the 6.0 or 10 mLs of whole blood, depending on the collection tube size used, to prevent platelet aggregation engulfing the spheroids or MCCCs. The spiked spheroid blood solution was aspirated into a 10mL injection syringe and attached to a syringe pump. A flow rate of 100µL/minute was used to infuse the blood into our PDMS microfluidic chip.

An accurate spiked co-cultured spheroid count was noted and compared to the number of IF imaged spheroids captured on the chip to calculate a capture efficiency percentage. The 100µL/minute flow rate was utilized to ensure spheroid integrity while flowing through the chip. Immediately following processing, both Inlet and Outlet ports were sealed, and the microfluidic chip was stored at ambient temperature in PBS until ready for imaging of the captured MCCC's. A simplified depiction of the sample processing along with a combined fluorescence / brightfield image of a captured co-cultured spheroid is shown in S3 Fig.


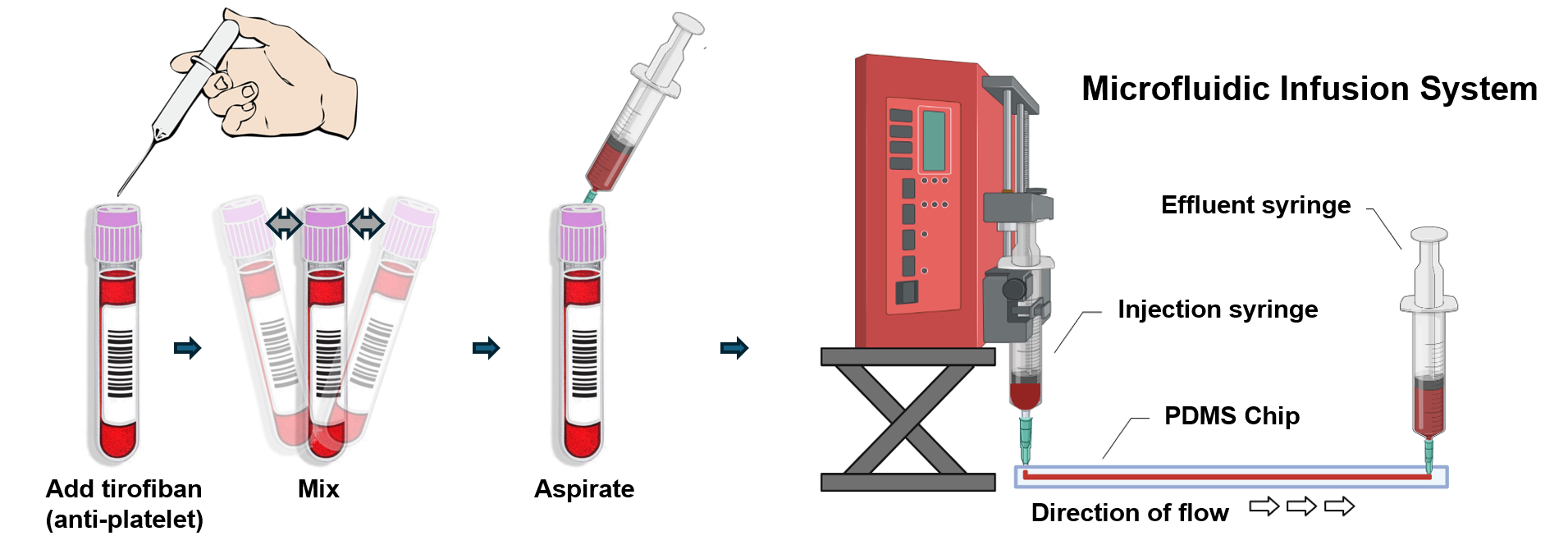


**S2 Fig. Schematic of sample processing**. A syringe containing an unprocessed patient blood sample is injected into microfluidic capture chip at a flow rate of 100μL/minute. Blood collection, transport and processing are maintained at ambient temperature. Effluent can be collected and stored for subsequent multiplexed analyses (ctDNA, exosome, etc.).

**A B**


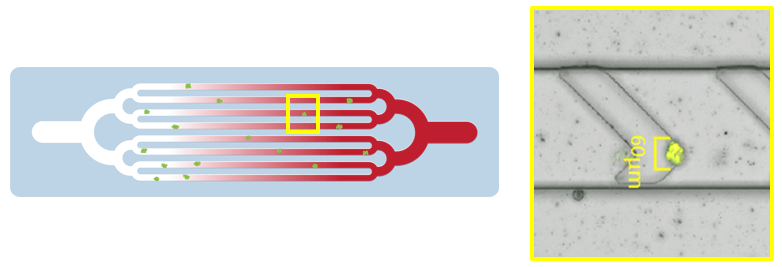


**S3 Fig. Simplified illustration of the sample processing and an image of a co-cultured spheroid.** **A**. An illustration depicting whole blood processing where MCCCs are captured on our Smart-Coating™ within the channel walls of our microfluidic chip, while all remaining RBCs and WBCs are eluted. **B**. Is a composite fluorescent and brightfield microscopy image of a captured co-cultured HCC827 spheroid.

**RNA-seq process and bioinformatics analysis**

llumina sequencing was performed on RNA extracted from the captured MCCCs. Three RNA aliquots of the LC6 sample were processed for library preparation using the SMART-Seq® HT Kit which combines both cDNA synthesis and amplification (TaKaRa Cat.#634455) plus Nextera® XT Sample Prep Kit (Illumina Cat.#15032354) for tagmentation. Standard Illumina bioinformatics analysis was used to generate fastq files, followed by quality assessment [MultiQC v1.7 https://multiqc.info/]. All downstream bioinformatic analyses were done with R/Bioconductor packages. ‘Rsubread’ v2.10.4 was used for mapping to the hg38 genome and creating a matrix of RNA-Seq counts. Next, a DGElist object was created with the ‘edgeR’ package v3.38.1 [https://doi.org/10.1093/bioinformatics/btp616]. After filtering-out lowly expressed genes, normalization for composition bias was performed using the TMM method (trimmed mean of M-values) as proposed by Robinson and Oshlack.[15] Briefly, the total RNA production cannot be estimated directly, since we do not know the expression levels of every gene. However, the relative RNA production of two samples, essentially a global fold change, can more easily be determined. TMM is a simple yet robust way to estimate the ratio of RNA production under the assumption that the majority of genes are not differentially expressed. In this method the M-values (or average expression stability values) are weighted according to inverse variances, as computed by the delta method for logarithms of binomial random variables. Of note, normalization factors obtained with the TMM method were not used to transform the data but were instead included in the statistical model in the form of scaling factors for the library sizes. Following normalization, genewise exact tests were computed for differences in the means between groups, and differentially expressed genes (DEGs) were extracted based on an FDR-adjusted p value < 0.05.

For RNA-seq integration with datasets GSE74639, GSM18494, and GSM18950, count data corresponding to each of these datasets was downloaded directly from the GEO repository and combined (separately for each dataset) with the common features of the MCCC counts in a single DGElist object. After preprocessing with 'edgeR', batch correction was performed with the 'removeBatchEffect' function of the 'limma' package, using the dataset of origin as the batch variable. This function fits a linear model to the data, including both batches and regular treatments, then removes the component due to the batch effects. The output is a numeric matrix of log-expression values with batch effects removed that was used for downstream analyses. Visualization of normalized and batch-corrected gene expression data was done with the 'ggplot2' package.

**S1 Table. Key Resources Table**

| **Reagent or Resource** | **Source** | **Identifier** |
| --- | --- | --- |
| **Capture Antibodies / Coating Reagents** |  |  |
| Biotinylated anti-EGFR | Santa Cruz Biotechnology | Cat.# SC-120B |
| Biotinylated anti-MET | Cell Signaling Technology | Cat.# 64526BC |
| Biotinylated anti-HER3 | LS Bio | Cat.# LS-C87995 |
| Streptavidin | AAT Bioquest | Cat.# 16885 |
| Sodium Alginate-Pronova-UP-VLVG | NovaMatrix/IFF | Cat.# 4200501  <https://novamatrix.biz/store/pronova-up-vlvg/> |
| Hyaluronic acid-comestic grade | Resurrection Beauty | Cat.# High-MW-1800kDa  [https://stores.resurrectionbeauty.com/hyaluronic-acid-powder](https://www.amazon.com/Hyaluronic-Molecular-Hyaluronate-Hydrating-Ingredient/dp/B00WTEB9GY?ref_=ast_sto_dp) |
|  |  |  |
| **Staining Antibodies / Dyes** |  |  |
| Anti-CD44-FITC | Abeomics | Cat.# 10-7516-F |
| Anti-EGFR-AF594 | Santa Cruz Biotechnology | Cat.# SC-120-AF594 |
| Hoechst 33342 nuclear stain | Thermo Scientific | Cat.# H3570 |
|  |  |  |
| **Cell Lines** |  |  |
| NSCLC - HCC827 | ATCC | Cat.# CRL-2868  <https://www.atcc.org/products/crl-2868> |
| NSCLC - A549-GFP | Angio-Proteomie | Cat.# cAP-0097GFP  <https://www.angioproteomie.com/commerce/ccp23644-gfp-human-lung-5bnsclc5d-carcinoma-cells-28a54929-cap-0097gfp.htm> |
| Human foreskin fibroblasts-HFF-1 | ATCC | Cat.# SCRC-1041  <https://www.atcc.org/products/scrc-1041> |
|  |  |  |

**S2 Table Links to MCCC RNA-seq gene expression datasets**

top.genes.log2.xlsx: highly expressed genes >1000 counts

Excel tables with the normalized data (counts in base 2 logarithmic scale), before and after filtering out genes with less than 10 counts in average across the 3 replicates.

logcpm_unfiltered.xlsx:

logcpm_filtered.xlsx:

**S3 Table**

**Drug Gene Interaction Data Base results for AKR1B1, ALK, CD274, EGF, EML4, ERBB3, ERBB4, TP53, VEGFC and links to published RNA-seq datasets.**

DGIDdb_Gene_Interaction_Results.xlsx

The published gene expression datasets downloaded from the Gene Expression Omnibus (GEO) website via the following links:

GSM18949 <https://www.ncbi.nlm.nih.gov/geo/query/acc.cgi?acc=GSM18949>

GSM18950 <https://www.ncbi.nlm.nih.gov/geo/query/acc.cgi?acc=GSM18950>

GSE74639 <https://www.ncbi.nlm.nih.gov/geo/query/acc.cgi?acc=GSE74639>

References

1. Kapeleris J, Zou H, Qi Y, Gu Y, Li J, Schoning J, et al. Cancer stemness contributes to cluster formation of colon cancer cells and high metastatic potentials. Clin Exp Pharmacol Physiol. 2020;47(5):838-47.

2. Liu X, Taftaf R, Kawaguchi M, Chang YF, Chen W, Entenberg D, et al. Homophilic CD44 Interactions Mediate Tumor Cell Aggregation and Polyclonal Metastasis in Patient-Derived Breast Cancer Models. Cancer Discov. 2019;9(1):96-113.

3. Kawaguchi M, Dashzeveg N, Cao Y, Jia Y, Liu X, Shen Y, et al. Extracellular Domains I and II of cell-surface glycoprotein CD44 mediate its trans-homophilic dimerization and tumor cluster aggregation. J Biol Chem. 2020;295(9):2640-9.

4. Rodrigues P, Vanharanta S. Circulating Tumor Cells: Come Together, Right Now, Over Metastasis. Cancer Discov. 2019;9(1):22-4.

5. San Juan BP, Garcia-Leon MJ, Rangel L, Goetz JG, Chaffer CL. The Complexities of Metastasis. Cancers (Basel). 2019;11(10).

6. Baaten BJ, Tinoco R, Chen AT, Bradley LM. Regulation of Antigen-Experienced T Cells: Lessons from the Quintessential Memory Marker CD44. Front Immunol. 2012;3:23.

7. Teriete P, Banerji S, Noble M, Blundell CD, Wright AJ, Pickford AR, et al. Structure of the regulatory hyaluronan binding domain in the inflammatory leukocyte homing receptor CD44. Mol Cell. 2004;13(4):483-96.

8. Ponta H, Sherman L, Herrlich PA. CD44: from adhesion molecules to signalling regulators. Nat Rev Mol Cell Biol. 2003;4(1):33-45.

9. Schnoor M, Alcaide P, Voisin MB, van Buul JD. Crossing the Vascular Wall: Common and Unique Mechanisms Exploited by Different Leukocyte Subsets during Extravasation. Mediators Inflamm. 2015;2015:946509.

10. Siegelman MH, Stanescu D, Estess P. The CD44-initiated pathway of T-cell extravasation uses VLA-4 but not LFA-1 for firm adhesion. J Clin Invest. 2000;105(5):683-91.

11. Brennan FR, O'Neill JK, Allen SJ, Butter C, Nuki G, Baker D. CD44 is involved in selective leucocyte extravasation during inflammatory central nervous system disease. Immunology. 1999;98(3):427-35.

12. Castro-Giner F, Aceto N. Tracking cancer progression: from circulating tumor cells to metastasis. Genome Med. 2020;12(1):31.

13. Chaffer CL, Goetz JG. CD44 Orchestrates Metastatic Teamwork. Dev Cell. 2018;47(6):691-3.

14. Shaner SW, Allen JK, Felderman M, Pasko ET, Wimer CD, Cosford NDP, et al. Design and production of a novel microfluidic device for the capture and isolation of circulating tumor cell clusters. AIP Advances. 2019;9(6):065313.

15. Robinson MD, Oshlack A. A scaling normalization method for differential expression analysis of RNA-seq data. Genome Biol. 2010;11(3):R25.
