# Supplementary figures and images for: Capture of circulating metastatic cancer cell clusters from lung cancer patients can reveal unique genomic profiles and potential anti-metastatic molecular targets: A proof-of-concept study
Source: PLoS One. 2024 Jul 31;19(7):e0306450. doi: 10.1371/journal.pone.0306450 (PMC11290651; doi:10.1371/journal.pone.0306450)

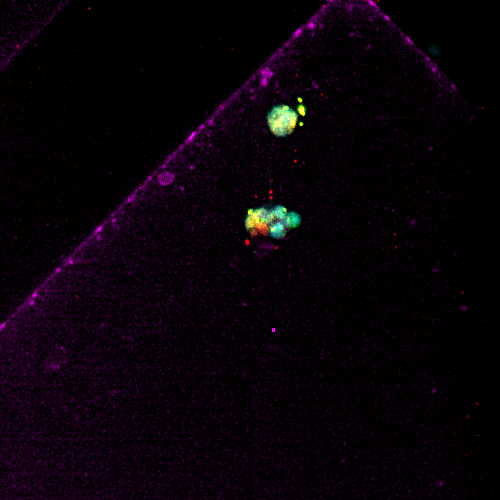

Supplement: S2 File — (PNG) [file pone.0306450.s002.png]
