# Supplementary material for: Correction: Capture of circulating metastatic cancer cell clusters from lung cancer patients can reveal unique genomic profiles and potential anti-metastatic molecular targets: A proof-of-concept study
Source: PLoS One. 2025 Mar 25;20(3):e0321150. doi: 10.1371/journal.pone.0321150 (PMC11936155; doi:10.1371/journal.pone.0321150)
Supplement: S2 File — (PDF) [file pone.0321150.s002.pdf]

PLOS ONE

RESEARCH ARTICLE

Capture of circulating metastatic cancer cell clusters from lung cancer patients can reveal unique genomic profiles and potential anti-metastatic molecular targets: A proof-of-concept study

Kourosh Kouhmareh[1¤a], Erika Martin[1], Darren Finlay[2], Anukriti Bhadada[3¤b], Hector Hernandez-Vargas[4], Francisco Downey[3¤c], Jeffrey K. Allen[3], Peter Teriete[3,5]*

1 PhenoVista Biosciences, San Diego, CA, United States of America, **2** National Cancer Institute Cancer Center, Sanford Burnham Prebys Medical Discovery Institute, La Jolla, CA, United States of America, **3** TumorGen Inc., San Diego, CA, United States of America, **4** Centre Léon Bérard, Lyon, France, **5** IDEAYA Biosciences, South San Francisco, CA, United States of America

¤a Current address: Pathology Department, University of California San Diego, La Jolla, CA, United States of America
¤b Current address: NanoCellect Biomedical, Inc., San Diego, CA, United States of America
¤c Current address: Department of Bioengineering, University of California San Diego, La Jolla, CA, United States of America
* pteriete@tumorgen.net

Check for updates

OPEN ACCESS

Citation: Kouhmareh K, Martin E, Finlay D, Bhadada A, Hernandez-Vargas H, Downey F, et al. (2024) Capture of circulating metastatic cancer cell clusters from lung cancer patients can reveal unique genomic profiles and potential anti-metastatic molecular targets: A proof-of-concept study. PLoS ONE 19(7): e0306450. https://doi.org/10.1371/journal.pone.0306450

Editor: Dominique Heymann, Universite de Nantes, FRANCE

Received: February 28, 2024

Accepted: June 18, 2024

Published: July 31, 2024

Copyright: © 2024 Kouhmareh et al. This is an open access article distributed under the terms of the Creative Commons Attribution License, which permits unrestricted use, distribution, and reproduction in any medium, provided the original author and source are credited.

Data Availability Statement: All relevant data are within the manuscript and its Supporting Information files.

Funding: 1. SBIR Grant from the National Cancer Institute 1R43CA261362-01 a. https://reporter.nih.gov/project-details/10255872 2. CCSG Grant P30CA23100 supporting the Biorepository and Tissue Technology Shared Resource at UCSD

Abstract

Metastasis remains the leading cause of cancer deaths worldwide and lung cancer, known for its highly metastatic progression, remains among the most lethal of malignancies. Lung cancer metastasis can selectively spread to multiple different organs, however the genetic and molecular drivers for this process are still poorly understood. Understanding the heterogeneous genomic profile of lung cancer metastases is considered key in identifying therapeutic targets that prevent its spread. Research has identified the key source for metastasis being clusters of cells rather than individual cancer cells. These clusters, known as metastatic cancer cell clusters (MCCCs) have been shown to be 100-fold more tumorigenic than individual cancer cells. Unfortunately, access to these primary drivers of metastases remains difficult and has limited our understanding of their molecular and genomic profiles. Strong evidence in the literature suggests that differentially regulated biological pathways in MCCCs can provide new therapeutic drug targets to help combat cancer metastases. In order to expand research into MCCCs and their role in metastasis, we demonstrate a novel, proof of principle technology, to capture MCCCs directly from patients' whole blood. Our platform can be readily tuned for different solid tumor types by combining a biomimicry-based margination effect coupled with immunoaffinity to isolate MCCCs. Adopting a selective capture approach based on overexpressed CD44 in MCCCs provides a methodology that preferentially isolates them from whole blood. Furthermore, we demonstrate a high capture efficiency of more than 90% when spiking MCCC-like model cell clusters into whole blood. Characterization of the captured MCCCs from lung cancer patients by

Moores Cancer Center the funders had no role in study design, data collection and analysis, decision to publish, or preparation of the manuscript.

Competing interests: Jeffrey K. Allen is the President/Founder of TumorGen and holds shares of the company as well as Peter Teriete who is the Co-Founder/Scientific Advisor of TumorGen and also holds shares of the company. Please note that holding these interests does not alter our adherence to PLOS ONE policies on sharing data and materials.

immunofluorescence staining and genomic analyses, suggests highly differential morphologies and genomic profiles. This study lays the foundation to identify potential drug targets thus unlocking a new area of anti-metastatic therapeutics.

Introduction

Significance of metastatic cancer cell clusters

Metastasis remains the primary cause of cancer-related deaths. A recently published clinical analysis identifies metastasis as the cause of death in 50.2% to 90.4% of cases, depending on the origin of the primary tumor [1]. The resulting high metastatic death rates indicate that increased focus on anti-metastatic therapy is urgently needed. Many cancer types have a demonstrated propensity to establish distant metastases among a defined set of organs.

Non-small cell lung cancer (NSCLC), a major malignancy with high mortality, typically metastasizes to bone, followed by brain, liver and adrenal glands [2]. Lung cancer is a prime example of mortality driven by distant metastases to many vital organs [3, 4]. However, the mechanism driving the spread, in particular cancer cell transportation and immune evasion is not well understood. The inability to isolate and profile circulating metastatic cells constitutes a major challenge in beginning to understand these pathways.

Over the past decade, tremendous strides in developing better therapies via molecularly targeted and immune-checkpoint inhibitor treatments, have improved patient outcomes [5]. However, similar significant gains have not been observed for metastatic disease [6]. A key factor in this discrepancy is the complexity of cancer metastasis. Evidence has shown that signaling pathways driving metastatic lesions are often different than found within the primary tumor [7]. A recent publication from Anderson *et al.*, representing the Cancer Research UK and Australia Metastasis Working Group stated: "The standard cancer drug discovery and development pathway, including that for molecularly targeted and immunotherapies, generally ignores the ability of experimental medicines to inhibit metastasis." The authors went on to highlight: "To treat metastasis effectively, we must inhibit fundamental metastatic processes and develop specific preclinical and clinical strategies that do not rely on primary tumor responses." [8]

Over the last decade, extensive research into individual circulating tumor cells (CTCs), has begun to shed light on tumor proliferation however, it has not produced a unique CTC genomic profile reflecting metastatic pathways across multiple tumor types [9]. Adding further complexity in understanding these mechanisms, the majority of individual cancer cells in circulation undergo anoikis or are eliminated by the immune system [10]. However, an important smaller subset of CTCs circulates in the bloodstream as a cluster of two or more cells. Compelling evidence has demonstrated that CTC clusters have a significantly higher tumorigenic potential once they enter circulation as opposed to individual CTCs [11–13].

Regarding circulating clusters or metastatic clusters, publications have referred to them with different nomenclature such as: circulating tumor cell clusters (CTC clusters), [14] circulating tumor micro-emboli (CTMs), [15] clustered circulating cancer stem cells (cCSCs), [16] metastatic cancer cell clusters, [17] or by using a more general term called collective cell migration, [18] yet all share common characteristics such as a being composed of 2–50 cells with up to 100-fold higher metastatic potential than individual CTCs [14, 19]. Here we will use the terms MCCCs and CTC clusters interchangeably to refer to these circulating, highly metastatic entities.

A number of recent studies have begun to elucidate the phenotypic composition of these MCCCs and have shown them as either homotypic, composed of all cancer cells, or heterotypic, containing multiple cell types including cancerous plus non-cancerous cells [20–22]. The MCCC phenotype possesses an adaptive mechanism that enhances their survival in the harsh bloodstream environment contributing to their metastatic potential [23–26]. Specifically, heterotypic MCCCs demonstrate an increased ability to evade immune surveillance which is postulated to be a result of cancer cell and immune cell communication within the cluster [27–29]. The metastatic cancer cell clustering phenomenon is also thought to mitigate an immune response from natural killer (NK) cells, due to surface receptors, in addition to intercellular communication present within CTC clusters [30, 31].

New drug therapies, focused on MCCCs, will have the potential to dramatically reduce metastasis and improve patient survival. A landmark paper published in 2019 by Aceto *et al*. demonstrated that targeting MCCCs significantly reduces metastasis in an orthotopic breast cancer model in mice [32]. Genomic analysis of breast cancer clusters from patients revealed 10 differentially regulated pathways unique to metastatic clusters [32]. Cardiac glycosides, an existing class of drugs targeting one of those pathways, was evaluated in an *in vivo* animal model. Treatment with one of the drugs (Ouabain) reduced the number of CTC clusters by 60% and decreased metastasis to the lungs and other organs by 80-fold. An intriguing aspect from this work is that the Ouabain dosage levels utilized are not cytotoxic and only disrupted the MCCCs leading to a reduced metastatic index *in vivo*. This study strongly supports the necessity to characterize the MCCCs from patients, to identify previously unseen therapeutic targets and ultimately develop novel drugs thus opening a completely new paradigm in anti-metastatic therapy.

Current tumor cell isolation methods from whole blood

Insights into how solid tumors propagate to specific pre-metastatic sites is necessary to develop focused therapeutic approaches that can prevent metastasis. While the importance of MCCCs has been well established, technologies to capture and characterize the MCCCs remain problematic and has limited research into this field.

Most existing technologies used to capture CTC clusters or MCCCs, rely on systems designed to capture single CTCs. Many technologies are based on a size filtration mechanism for capture, relying upon the premise that individual CTCs are larger than most leukocytes [33, 34]. Using size filtration, patient blood samples are processed and then sorted based on the individual cell diameter. Whole blood consists of three main components: platelets, red blood cells (RBCs), and white blood cells (WBCs). White blood cells, which are the largest of the three components, are generally 12–15μm in size. Composed of multiple cells, MCCCs are presumed larger than WBCs when present in whole blood, thus would be isolated by size for further profiling. Although the main benefit of size-based technology is that it's label-free and requires little pre-processing, it has major limitations when it comes to successfully identifying and isolating several types of individual CTCs and MCCCs. It is possible for smaller MCCC's and many CTC's to be falsely binned as white blood cells, since the average cell diameters of CTC's fall within the 10–15μm range [35–37]. A recent review also stated that microfluidic channel size can induce critical flow velocities resulting in shearing and disaggregation of CTC clusters [19, 38]. Another review by Amintas *et al*. concluded that individual CTC isolation techniques are typically not compatible with CTC cluster isolation. They identified three key requirements stating: "Future developments should aim towards methods that allow phenotypic, molecular, and even functional analyses" [39].

New metastatic cancer cell cluster isolation method

We have developed a highly selective capture technology with a positive capture bias toward MCCCs by specifically exploiting multiple features unique to MCCCs. Their size and thus specific margination behavior during microfluidic flow conditions, [40, 41] as well as overexpression of CD44 on the surface of most cell types found in MCCCs can be utilized for capture from whole blood [42]. The CD44 surface expression is an abundant marker of MCCCs, upregulation of which closely correlates to their metastatic potential [43–47]. Our selective capture approach is based on a biomimetic CD44-induced cell cluster margination effect combined with immobilized capture antibodies coated within the inner walls of a microfluidic chip.

We are reporting on the success of our highly selective capture platform, focused on lung cancer, that preferentially isolates cell clusters predisposed to metastasis. Our technology allows routine and efficient capture of MCCCs providing insights into their biology as well as elucidation of new anti-metastatic therapeutic targets. Ultimately, this will expedite the development of new drugs capable of disrupting clusters and/or cluster formation, thus reducing metastasis.

Materials and methods

Microfluidic chip manufacturing and coating procedure

Design, manufacturing, and coating of the microfluidic chips has been published previously [48]. Production of the microfluidic chips used for this study were outsourced to a commercial partner, µFluidix (Scarborough Ontario, Canada). Each chip was produced from two separate layers of polydimethylsiloxane (PDMS) which were then precision aligned to create our open channel microfluidic chips. The design parameters include 32 individual channels (100µm H x 200µm W each) along with a 'ceiling' feature composed of alternating patterns of recessed chevrons, 50µm high which induces chaotic flow [48].

Ultrapure pharmaceutical grade alginate (Novamatrix; Sandvika, Norway) used for the hydrogel substrate of the Smart-Coating™, was derivatized with streptavidin (AAT Bioquest, Cat.#16885) molecules using carbodiimide chemistry via the carboxylic groups of the alginate sugar residues [49].Coating of the microfluidic chips with a streptavidin derivatized, calcium (Ca^{+2}) cross-linked alginate hydrogel was performed as described previously [48] A secondary coating consisting of a mixture of biotinylated hyaluronic acid and biotinylated monoclonal antibodies (mAbs) was added to the alginate hydrogel. The secondary coating was prepared using cosmetic grade hyaluronic acid (HA) powder (Resurrection Beauty; Cat.# High-MW-1800kDA) that was biotinylated and Cy5-labeled (1:1:1 molar ratios) using carbodiimide chemistry via the carboxylic groups of the glucuronic acid moiety using Biotin-Hydrazide, (Thermo Scientific, Cat.# 21340; and Cy5-Hydrazide from ApexBio, Cat.# A8145). The labeled HA plus a combination of biotinylated monoclonal antibodies was mixed at a 1:16 ratio into phosphate buffered saline (PBS, 1X Caisson Labs PBL01-500ML) along with 0.1% Tween-20. The 4.5 mL mixture was infused onto the alginate coated chip at 200 uL/min and the chip was subsequently washed with 2.5 mL of PBS at 2.5 mL/min. After the second coating was complete chips were stored in a sealed conical tube containing PBS at room temperature until needed. The S1 Fig in S1 File shows a successful coating of our chip with the fluorescently labeled biotinylated hyaluronic acid-Cy5 (bHA-Cy5) bound to the streptavidin derivatized, cross-linked alginate hydrogel. The fluorescent image of S1 Fig in S1 File also shows a captured model MCCC which is a heterotypic spheroid composed of NSCLC A549-GFP (green) co-cultured with human foreskin fibroblast (red, HFFs) cells.

Three surface epitopes, EGFR (ERBB1), MET and HER3 (ERBB3) were selected based on published reports that they are typically expressed on the surface of lung cells [50–52]. Three commercially available antibodies that bind to these epitopes were selected: biotinylated anti-EGFR (Santa Cruz Biotechnology, Cat.# SC-120B) biotinylated anti-MET (Cell Signaling Technology, Cat.#64526BC) and biotinylated anti-HER3 (LS Bio Cat.#LS-C87995). All purchased antibodies were tested to confirm cell surface binding on established NSCLC cell lines, HCC827 (ATCC, CRL-2868) and A549 (Angio-Proteomie cAP-0097GFP) by immunofluorescence (IF). Cell line authentication was based on vendor information provided. See S1 File.

Experiments to assess the microfluidic chip capture efficiency were conducted with multicellular heterotypic spheroids, generated using established cell lines co-cultured with the non-cancerous human foreskin fibroblasts, HFF cells, as cancer associated fibroblast surrogates. A known number of model NSCLC MCCCs using co-cultured spheroids (1:1 ratio of NSCLC HCC827 and non-cancerous human foreskin fibroblasts, HFF cells) were spiked into normal donor whole blood samples and processed in triplicate. Results comparing zero antibodies, (Smart-Coating™ biotinylated hyaluronic acid / alginate hydrogel only) to 1 antibody (anti-EGFR) and 3 antibodies, (anti-EGFR, anti-MET and anti-HER3 mAbs combined) were performed. See the S1 File Materials and methods section, along with S2 and S3 Figs in S1 File for additional details on co-cultured spheroid preparation, culture times, staining procedures, and blood processing schematic.

NSCLC patient whole blood processing

A total of eight patient samples (mean age male [n = 3]: 70.9, ssd 1.1; female [n = 5]: 57.1, ssd 1.2) were collected from the University of California, San Diego (UCSD) Moores Cancer Center, an NCI-Designated Comprehensive Cancer Center. Biospecimens were collected by the Moores Cancer Center Biorepository from consenting patients under a University of California, San Diego Human Research Protections Program Institutional Review Board approved protocol (HRPP# 181755). Biorepository subjects written consent is maintained in the Biorepository archives. Patients were enrolled into the study from July 7th through August 4th, 2022. All patients received comprehensive study information from the UCSD biorepository (BR) coordinator and provided written consent as described in section D of the IRB approval form. Patients previously diagnosed with non-small cell lung cancer (NSCLC), at stages III-A, III-B, III-C, IV-A, IV-B agreeing to participate in a study, graciously provided whole blood samples. Non-identifying patient information was collected for each blood sample, including age/gender, confirmed diagnosis, and cancer stage, at the time of blood draw. Patient's sex and demographic information was not considered as inclusion or exclusion criteria for this study. Future studies involving a higher number of patients will consider these factors to help understanding existing disparities. Sample processing was blinded to patient information.

Collection tubes were treated with EDTA and Tirofiban to prevent coagulation and platelet aggregation respectively [53]. Samples were transported on the same day at ambient temperature and processed through our microfluidic system within 8 hours. In an effort to preserve sample integrity to the fullest extent, no red blood cell (RBC) lysis or whole blood pre-processing was performed, since reports of CTC loses have been noted due to pre-treatment methods [54, 55]. The entirety of the patient collected sample was aspirated into a 10mL syringe, attached to a syringe pump and processed through our coated microfluidic chip at a flow rate of 100μL/minute.

Flow was not disturbed until the entire sample had been infused through the microfluidic chip. After processing our patient blood sample, the chip was flushed with PBS at a rate of 100μL/minute for 5 minutes to remove any remaining unbound blood or white blood cells. A

4% formaldehyde, methanol-free (PFA) solution was then pumped through at 100μL/minute followed by another PBS wash for 5 minutes. Five of the patients' samples were processed for immunofluorescence imaging and analysis. Cells were stained with a 10 minute flush of our staining palette consisting of Hoechst 33342 live nuclear stain, (Thermo Scientific Cat.# H3570), anti-CD44-FITC (Abeomic Cat.# 10-7516-F) and anti-EGFR-AF594 (Santa Cruz Biotechnology Cat.# SC-120-AF594) all diluted in PBS, followed by a 5 minute PBS wash, at the 100μL/minute flow rate.

Our processed chip was imaged across all flow channels using the Yokogawa CQ1 scanner by stitching together 936 fields captured using a 10x objective. Chips were scanned for Brightfield, Hoechst staining (405nm), CD44 (488nm), EGFR (594nm), and our bHA-Cy5 chip coating (647nm). A relative increase of CD44 and EGFR fluorescent signal against background, including well-defined nuclei morphology, were essential gating parameters used to identify potential MCCCs. Using our 10X stitched composite image as a map for locating potential MCCC candidates, we re-imaged at pre-selected points using a 40x objective to confirm MCCC identification. Using a relatively strict inclusion criteria, any irregular nuclei illuminated via the Hoechst signal even if coupled with positive CD44 and EGFR signal were rejected as MCCC candidates. Furthermore, at least 3 individual nuclei had to be discernable in order to be classified as a candidate MCCC.

NSCLC whole blood processing for RNA-seq analysis

Three additional patient LC6, LC7, LC8 (selected at random, see Table 1) whole blood samples were processed for RNA-sequencing analysis. Following whole blood infusion, a modified staining protocol was used to reduce imaging time to enhance RNA stability. The chips were flushed with Hoechst 33342 stain (without adding the anti-EGFR and anti-CD44 mAbs), at 100uL/min. for 15 minutes. The Hoechst stain was flushed out using a DMEMs-phenol red media (Thermo Fisher, Cat.# 11965084) at 100uL/min. for 5 minutes..

The Inlet and Outlet ports were sealed and imaged using fluorescence microscopy. Brightfield and 405nm images were collected to allow visualization of the captured MCCC nuclei. Post imaging, RNA lysis / stabilization reagent (TaKaRa, Cat. # STO948) was flushed through the microfluidic chip and incubated at zero flow for 15 minutes. Following lysis, fractions were collected at the Outlet port every 15 seconds providing a total of 8 fractions, each with an approximate volume of 25μL. Fractions were immediately frozen at -20˚C and sent for RNA sequencing analysis.

One of the patient samples, LC6, was submitted for RNA-seq analysis. This sample corresponded to a 64-year-old female patient, who was diagnosed by transthoracic biopsy with a TTF-1 positive Stage IV lung adenocarcinoma that had metastasized to the brain. Patient LC6 was negative for any EGFR mutations, is a non-smoker, and was on Brigatinib therapy at the time of blood collection. Three RNA lysis aliquots of the LC6 sample were processed and library preparation completed on the 3 aliquots.

RNA-seq process and bioinformatics analysis

llumina sequencing was performed on RNA extracted from the captured MCCCs. Three RNA aliquots of the LC6 sample were processed for library preparation using the SMART-Seq® HT Kit which combines both cDNA synthesis and amplification (TaKaRa Cat.#634455) plus Nextera® XT Sample Prep Kit (Illumina Cat.#15032354) for tagmentation. Standard Illumina bioinformatics analysis was used to generate fastq files, followed by quality assessment. [MultiQC v1.7 https://multiqc.info/]. The DupRadar plot in the MultiQC report confirmed linear amplification. Fastq files displayed Phred Quality scores ranging from 31.64 to 33.39, along

Table 1. Basic patient information and the number of MCCCs confirmed.

Patient	Age/ Gender	Diagnosis	Stage	# Clusters Detected	Detection Method
LC1	82 / M	lung adenocarcinoma	IVb	2	MCCC detection by bright field microscopy and confirmation by immunofluorescence staining for CD44, EGFR, Hoechst
LC2	53 / F	squamous cell lung carcinoma	IV	1	
LC3	59 / F	metastatic lung adenocarcinoma	IV	2	
LC4	65 / M	squamous cell lung carcinoma	IV	1	
LC5	67 / M	non-small cell lung carcinoma	IIIa	3	
LC6	64 / F	lung adenocarcinoma	IV	MCCC were not IF stained to preserve RNA quality	MCCC detection by bright field microscopy, samples processed for genomic analysis by RNA-seq.
LC7	46 / F	non-small cell lung cancer	IV		
LC8	66 / F	non-small cell lung cancer	IV		
Normal Blood Donors	54 / F	N/A	N/A	0	Neg. control
	68 / M			0	

https://doi.org/10.1371/journal.pone.0306450.t001

with high alignment (~95%) yet low gene assignment (~5% rates). Low assignment rates were explained by the small amount of MCCC DNA used in library preparation. All downstream bioinformatic analyses were done with R/Bioconductor packages. 'Rsubread' v2.10.4 was used for mapping to the hg38 genome and creating a matrix of RNA-Seq counts. Next, a DGElist object was created with the 'edgeR' package v3.38.1 [https://doi.org/10.1093/bioinformatics/btp616]. After filtering-out lowly expressed genes, normalization for composition bias was performed using the TMM method (trimmed mean of M-values) as proposed by Robinson and Oshlack [56]. After normalization, genewise exact tests were computed for differences in the means between groups, and differentially expressed genes (DEGs) were extracted based on an FDR-adjusted p value < 0.05.

For RNA-seq integration with datasets GSE74639, GSM18494, and GSM18950, count data corresponding to each of these datasets was downloaded directly from the GEO repository and combined (separately for each dataset) with the common features of the MCCC counts in a single DGElist object. After preprocessing with 'edgeR', batch correction was performed with the 'removeBatchEffect' function of the 'limma' package, using the dataset of origin as the batch variable. Visualization of normalized gene expression data was done with the 'ggplot2' package.

Datasets used for comparison to the LC6 technical replicates, were the following:

- GSE74639: RNA-Seq of human lung tumor circulating single cells (SCs, aka CTCs) and primary lung tumor cells (PTs) from an orthotopic lung xenograft model [57, 58].
- GSM18949 and GSM18950 are datasets identifying novel genes specific for human lung tissue [59, 60].

The published gene expression datasets were downloaded from the Gene Expression Omnibus (GEO) website. See S1 File for additional bioinformatic analysis details and the specific dataset links. Also, see the S1 Table in the S1 File section for the Key Resources Table which has vendor and catalogue numbers for the key reagents and cell lines utilized for this publication.

Results and discussion

Capture optimization

Our strategy was to develop a more 'selective' technology within a microfluidic system that would preferentially isolate and capture specific metastatic cell clusters rather than utilize a filtration-based or other size-based methodology. The premise was based on the extreme rarity of MCCCs among billions of red and white blood cells thus promoting the need to use an existing molecular biology process to enhance our cell selectivity. Leukocyte extravasation is a natural process involved with tissue damage, inflammation or infection.[61] Within the circulatory system, binding of CD44-presenting cells to endothelial hyaluronic acid (HA) is the first and essential step initiating margination, rolling, and extravasation [62]. Taking advantage of this existing biological principle, we designed and manufactured a microfluidic chip with a Smart-Coating™ technology that combines a tunable biomimetic and immuno-based dual capture approach [48]. An advantage of using our Smart-Coating™ is the ability to vary the number of monoclonal antibodies and biomimetic tethers which are integrated into the coating of the channel inner walls within the microfluidic chip. Immobilized streptavidin was derivatized on the cross-linked alginate hydrogel as previously described [48]. Antibodies with a biotin conjugate are easily bound to the streptavidin within the coating. Combinations of antibodies as well as biotinylated hyaluronic acid, which serves as a biomimetic tether, make up the second layer of our Smart-Coating. Since our platform is tunable, incorporating different biomimetic tethers and monoclonal antibodies enables the system to be adapted to other solid tumor cancer types.

Capture antibody selection for NSCLC

Identifying biomarkers on the surface of lung cells, that are relatively specific for lung tissue, is an important element of our platform. Multiple epitopes were utilized in our immuno-capture method, since this would likely isolate a larger diversity of MCCCs in circulation from different lung cancer patients. Experiments to assess the capture efficiency were conducted using multicellular co-cultured spheroids which were counted and spiked into normal whole blood samples. We added close to 100 spheroids per sample (4 mL) to have a sufficient spheroid count to quantify a wide range of capture efficiencies. This approach follows previously described methodologies [63]. The co-cultured spheroids generated using established NSCLC cell lines resemble the MCCCs found in patients' whole blood. As shown in Fig 1, using 3 capture antibodies resulted in a 90% capture efficiency, thus illustrating an advantage using multiple over a single capture antibody. Since utilizing a higher capture efficiency methodology will likely increase the probability of MCCC capture, 3 capture antibodies were used for subsequent NSCLC patient samples. The ability to capture co-cultured spheroids using the Smart-Coating alone, without any immobilized antibody, is likely due to the CD44 expression found on the co-cultured spheroids that have an avidity for the hyaluronic acid in our second coating [64, 65].

Patient samples

A total of 8 patient samples were collected from the UCSD Moores Cancer Center and processed through our Smart-Coated™ microfluidic chips that incorporated the 3 mAbs which demonstrated the highest capture efficiency. Two additional normal, healthy donor, patient whole blood samples were processed from a 54-year-old female and 68-year-old male, both of which revealed no confirmed MCCCs.

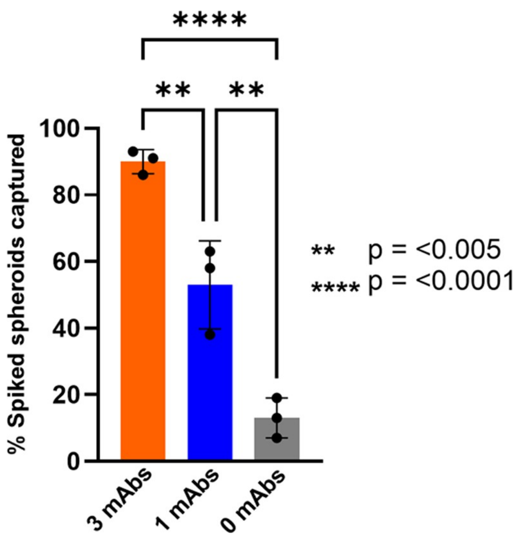

Fig 1. Capture efficiency comparison using varying numbers of tissue specific capture antibodies. The NSCLC co-cultured spheroids were spiked into freshly collected whole blood samples from normal donors. The exact spike count varied for each sample run but fell within the range of 71–91 spheroids. Post processing, NSCLC co-cultured spheroids were quantified via immunofluorescence. Samples were processed in triplicate. One-way ANOVA statistical analysis showed significant differences in capture efficiency between 0, 1, and 3 mAbs (** $P < 0.005$, *** $P < 0.0001$). 3 mAbs = anti-EGFR, anti-MET, anti-HER3. 1 mAbs = anti-EGFR. 0 mAbs = base Smart-Coating only with zero capture antibodies.

https://doi.org/10.1371/journal.pone.0306450.g001

Five of the patient samples, following processing on the chip, included immuno-fluorescence imaging for confirmation of the captured MCCCs using conjugated monoclonal antibodies as listed in Fig 2.

Detailed fluorescent image analysis of all microfluidic channels processed for each patient revealed MCCCs were identified and confirmed in all 5 samples. Basic patient information

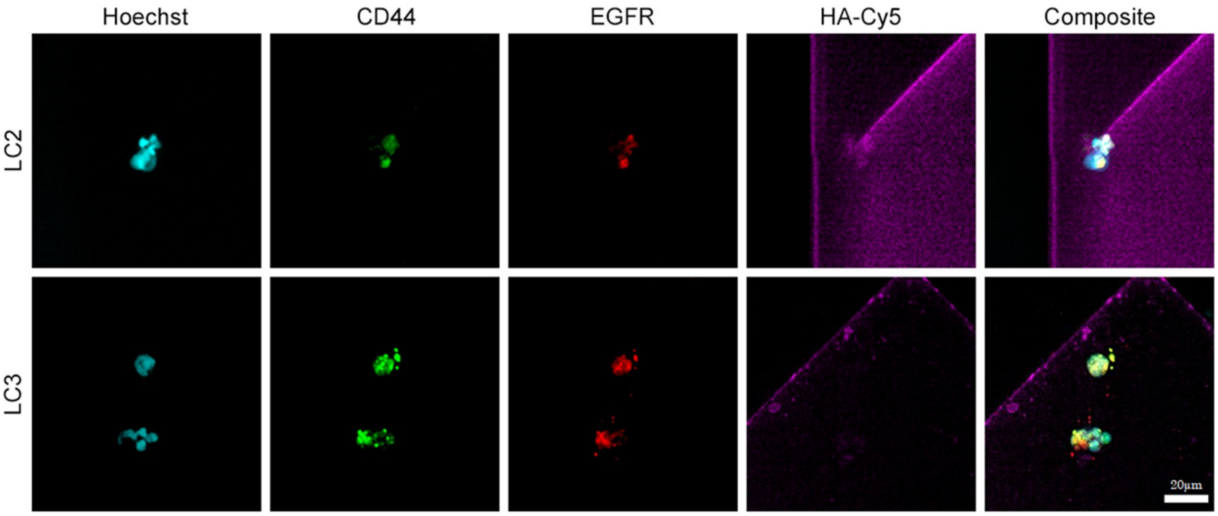

Fig 2. Representative 40X objective IF images of microfluidic chip captured NSCLC MCCCs from the whole blood of two NSCLC patients. Nuclei were stained with Hoechst 33342 (405nm blue) plus surface antigens with anti-CD44-FITC (488nm green) and anti-EGFR-AF594 (594nm red). The Smart-Coating™ is visualized via HA-Cy5 (647nm purple). The overlapping of both CD44 and EGFR fluorescence (yellow) in the composite image indicates cells expressing both markers. Two separate MCCCs were observed for patient LC3. Scale bar = 20μm for both LC2 and LC3 images.

https://doi.org/10.1371/journal.pone.0306450.g002

including diagnosis and disease staging plus detection method are listed along with the number of confirmed MCCCs, each consisting of a minimum of 3 morphologically distinct nuclei (Table 1).

The rationale for the selected surface antigens to confirm MCCCs is that the overexpression of both CD44 and EGFR is associated with cancerous lung cells [16, 43, 66]. Elevated fluorescent signal of anti-CD44 mAb conjugate and anti-EGFR(528) mAb combined with at least 3 distinct nuclei with well-defined nuclear morphology (Hoechst 33342) were essential gating parameters used to identify MCCCs. Observations of the captured MCCCs appear to show clusters with multiple nuclei that were relatively compact and not significantly larger than some single cell, non-cancerous monocytes. See Fig 2 [67, 68]. The observation of platelets associated with MCCCs, as reported in the literature, was not expected in our captured clusters since pretreatment with tirofiban disrupts platelet aggregation. Tirofiban treatment has been shown to increase CTC and MCCC surface antigen accessibility [53, 69, 70]. The remaining three NSCLC patient samples were processed with a modified protocol without immunofluorescence to help ensure RNA stability was maintained for subsequent ultra-low concentration bulk RNA sequencing analysis.

Imaged cell clusters were binned into three groups:

1. Hoechst (+), EGFR (-) and CD44 (-) Disregarded
2. Hoechst (+), CD44 (+) and EGFR (-) Disregarded
3. Hoechst (+), CD44 (+) and EGFR (+) Confirmed MCCC

Cells positive for only Hoechst and CD44 were presumed to be white bloods cells [71]. Any clusters with green or red positive staining without clear nuclear morphology via Hoechst staining were disregarded as potential MCCCs. Only clusters with all three stains, including 3 well defined nuclei were considered MCCCs. Examples of the confirmed NSCLC MCCCs captured from whole blood are shown in Fig 2. Note the heterogeneous shapes and sizes plus the multi-cellular composition as seen by the variation in fluorescence signal distribution among the cells in the MCCCs. A single MCCC was observed in patient LC2 which appears to have 5 nuclei. Also note the apparent compact cellular morphology of the clusters. Two separate, multi-cellular MCCCs were observed in patient LC3. The upper LC3 MCCC appears to have 3 separate nuclei and the lower MCCC has 4 nuclei present.

MCCC characterization via RNA-seq analysis

Determining the unique gene expression profiles represented within the MCCCs via RNA-seq analysis is crucial to begin learning more about the mechanisms of metastasis. Specifically, the ability to characterize the metastatic cancer cell clusters, captured while in transit, directly from a whole blood specimen will begin to shed light on the numerous signaling pathways involved in the metastatic cascade. Understanding the unique gene expression profile emanating from MCCCs found in NSCLC patient's whole blood is essential in discovering new anti-metastatic targets for therapeutic development. Previous research has reported MCCCs associated with fibroblasts, neutrophils, macrophages and platelets [70, 72, 73]. As mentioned above, heterotypic MCCCs have an immune survival advantage, thus all cell types should be included for sequencing to elucidate a complete profile [27–29]. The RNA-seq analysis produced from the captured MCCCs represents a profile from all captured cells, including both cancerous and non-cancerous cell types. Evidence of non-cancerous cells being captured was noted in the low-level expression of *PTPRC* (aka *CD45*), *CD163*, *CD99*, and *LYZ* which are common on monocytes and other WBCs (See, S1 Data) [74–76]. However, our use of a selective capture

methodology including rigorous washing minimizes the number of non-specifically bound cells that are unrelated to the MCCCs. Visualization via fluorescence microscopy of all microfluidic channels confirmed few other extraneous cells were present. [Data not shown]

Three additional patient samples with confirmed NSCLC diagnoses were processed through our microfluidic chips, using an abbreviated protocol, for the purpose of identifying gene expression profiles represented by the MCCCs. Following a modified processing method, MCCC capture was confirmed with Hoechst staining of the clustered nuclei. After imaging, the microfluidic chip was infused with RNA lysis/stabilization buffer, incubated and then aliquoted fractions were collected at the outlet port.

RNA-seq analysis was performed on the MCCCs captured from the 6th patient, designated LC6, who is a 64-year-old female with TTF-1 positive Stage IV lung adenocarcinoma. A 10mL whole blood sample was processed using our Smart-Coating™ microfluidic system. Post analysis imaging revealed multi-nucleated cell clusters (each with 3 or more nuclei based on Hoechst staining). RNA-seq analysis was performed on 3 separate lysate aliquots eluted from the chip processed from patient LC6. MCCC RNA sequencing data was of good quality, with high alignment rates to the human hg38 assembly (above 85% for the 3 technical replicates), Only a small fraction of reads (~ 5%), were assigned to genes, likely due to the low number of MCCC cells in the sample (S1 Data). Despite such a low assignment rate, approximately 7000 genes displayed at least 10 counts in the 3 MCCC samples (S2 Data). Moreover, 138 genes were highly expressed (over 1k reads) consistently across the 3 replicates (S3 Data).

Bioinformatic analysis of the RNA-seq data was used to confirm that the MCCCs captured originated from lung tissue. Expression levels were based on 6 well known genes found in lung cells from previously published datasets of human lung tissue, GSM18949 and GSM18950 [59, 77, 78]. The boxplots shown in Fig 3 also included normalized gene expression levels found in lung CTCs and lung primary tumor cells published elsewhere. This RNA-seq dataset (GSE74639) is comprised of lung tumor circulating single cells, designated (SCs) in the original manuscript, but also referred to here as CTCs as well as lung primary tumor cells (PTs) [57, 79].

Expression of five out of the six genes assessed were found within the MCCCs from patient LC6: *SFTPC*, *SCGB1A1*, *SLC34A2*, *SFTPB* and *CLDN18* thus confirming the tissue of origin for the captured MCCCs was lung. Another gene was identified within the captured MCCCs, *TTF-1* (aka *NKX2.1*), which is commonly used to aid in the diagnosis of primary pulmonary adenocarcinoma. The *NKX2.1* expression is found in 70–90% of non-mucinous adenocarcinoma subtypes [80]. The boxplot shown in Fig 3 reveals the expression of *TTF-1*(*NKX2.1*) in

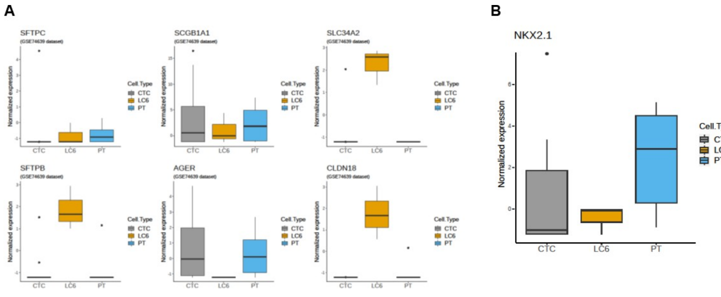

Fig 3. Normalized RNA-seq gene expression on a logarithmic y axis of 6 published genetic markers correlated to human lung tissue. (A) Plots shown are the comparisons of the MCCCs (patient LC6) to lung individual cells (CTCs) and lung primary tumor cells (PT) for those selected markers. The plots from 5 of the 6 markers selected confirm the lung cell origin of the MCCCs. **(B)** Boxplot showing the normalized gene expression of *NKX2.1* (aka *TTF-1*) of the MCCCs compared to both lung CTCs and lung primary tumor samples (PT). The detection of *TTF-1* from lung biopsy specimens is typically used to aid in the diagnosis of NSCLC.

https://doi.org/10.1371/journal.pone.0306450.g003

the MCCCs from patient LC6, which is consistent with the patient diagnosis obtained from the UCSD Moores Cancer Center, see Table 1. Data supports the finding that the MCCCs are from lung tissue and are adenocarcinoma cells.

Unfortunately, no RNA-seq data was available from our patients' primary tumor. Also, no datasets on aggregates or CTC clusters (aka MCCCs) from lung cancer patients are publicly available. Therefore, we utilized publicly available gene expression datasets from different NSCLC patients in comparison to our MCCC patient expression data. Using the described NSCLC RNA-seq datasets on primary tumor cells (PT) and individual CTCs (see Materials and methods) we performed an unsupervised clustering analysis comparing MCCCs with this data. Shown in Fig 4 is a Principal Component Analysis (PCA) of the integrated data sets comparing lung CTCs, lung PTs, and the 3 MCCC aliquoted samples (LC6). The PCA plot shows a very tight grouping of the LC6 aliquots, as well as discrete but more closely distributed grouping for the CTC and PT data from multiple patients.

In order to better visualize a unique gene expression profile, based on differentially expressed genes (DEGs) for the MCCCs, a heatmap was generated showing lung MCCCs relative to individual lung CTCs and lung primary tumor cells. The series GSE74639 RNA-seq dataset was utilized which contained 10 individually sequenced lung CTCs and 6 patient's lung primary tumor cells which were compared to the 3 aliquots of MCCCs from patient LC6 [57]. The MCCCs from patient LC6 display a discrete expression profile verses lung CTCs and PTs. The heatmap in Fig 5 shows that the MCCC profile shares some common gene expression among all 3 groups but also displays significant differences.

Even though a single MCCC data set is not ideal representing a unique expression profile, the published RNA-seq data sets from multiple patients in lung CTCs and primary tumor cells, segregated by cell type (CTCs vs. PTs), showed highly consistent profiles within a type among each patient. The clustering analysis of the single patient sample in our study is suggestive of a unique gene expression signature MCCCs harbor rather than individual patient differences. We believe that this reflects the contributions from the individual patient samples but also shows the fundamental differences between the origin of the cells sampled. Validation of this uniqueness will require extended patient sample testing incorporating autologous datasets

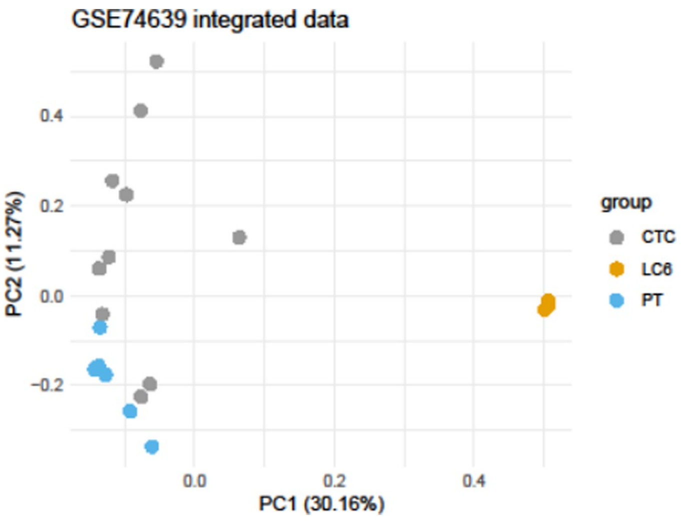

Fig 4. Principal Component Analysis (PCA) of the integrated data sets. The plot compares lung circulating tumor cells (CTC), lung primary tumor cells (PT) and the 3 MCCC aliquoted samples (LC6). The PCA plot of the patient's MCCCs sequence data are grouped separately compared to the other lung cancer cell types.

https://doi.org/10.1371/journal.pone.0306450.g004

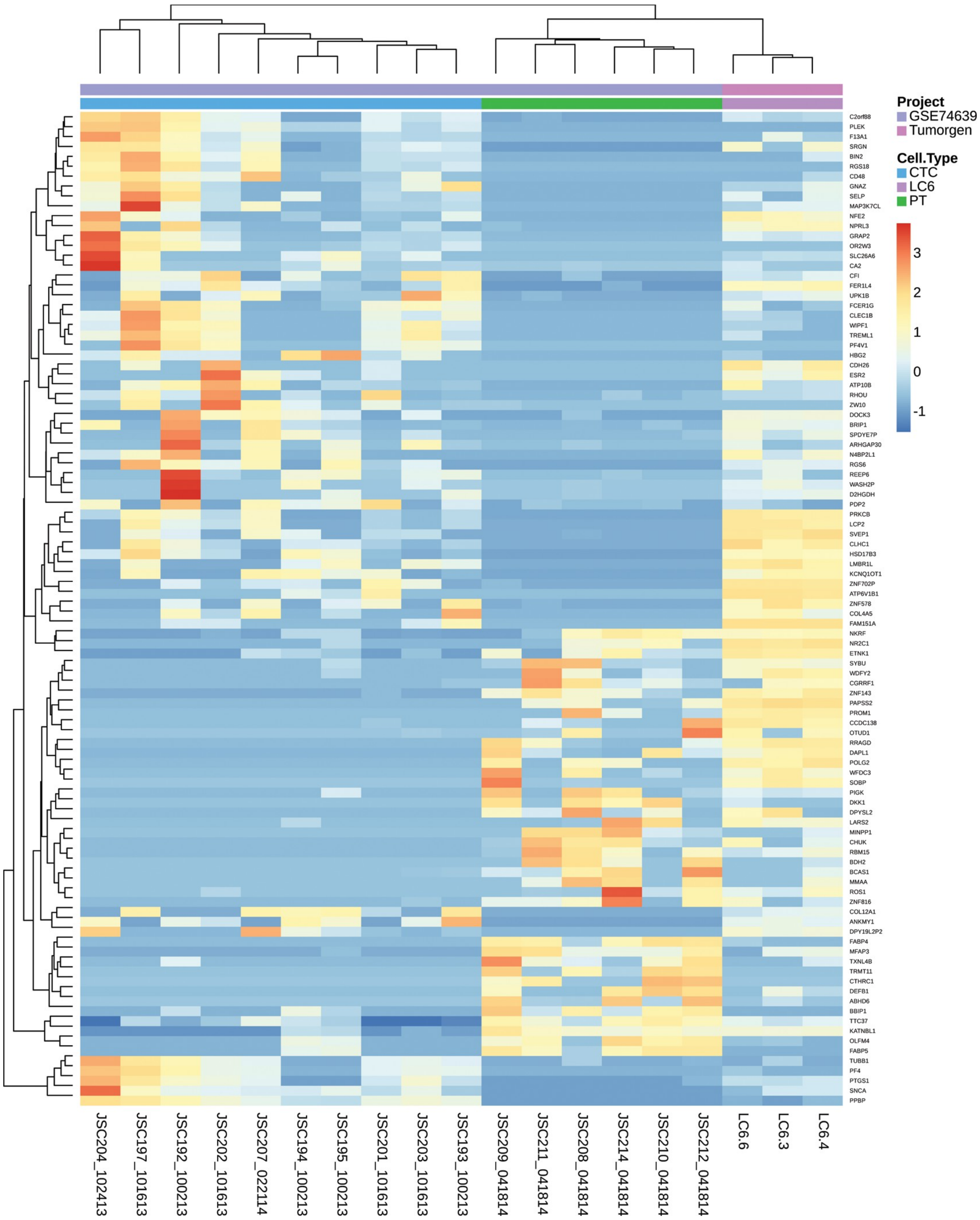

Fig 5. An RNA-seq heatmap generated using differentially expressed gene (DEGs) between PTs, CTCs and MCCCs. Although the MCCCs share some similarities between lung primary tumor cells (PTs) and individual lung circulating tumor cells (CTCs) from different patient studies, the DEG grouping of the MCCCs displays a unique profile.

https://doi.org/10.1371/journal.pone.0306450.g005

rather than comparisons to published CTC and primary tumor data. However, our proof-of-concept study clearly shows the ability to collect the necessary data to fully characterize MCCC RNA-seq profiles using our approach.

Fig 6A shows some examples of potentially actionable molecular targets expressed in the MCCC RNA-seq data. Cross-referencing of these targets with the Drug-Gene Interaction Data

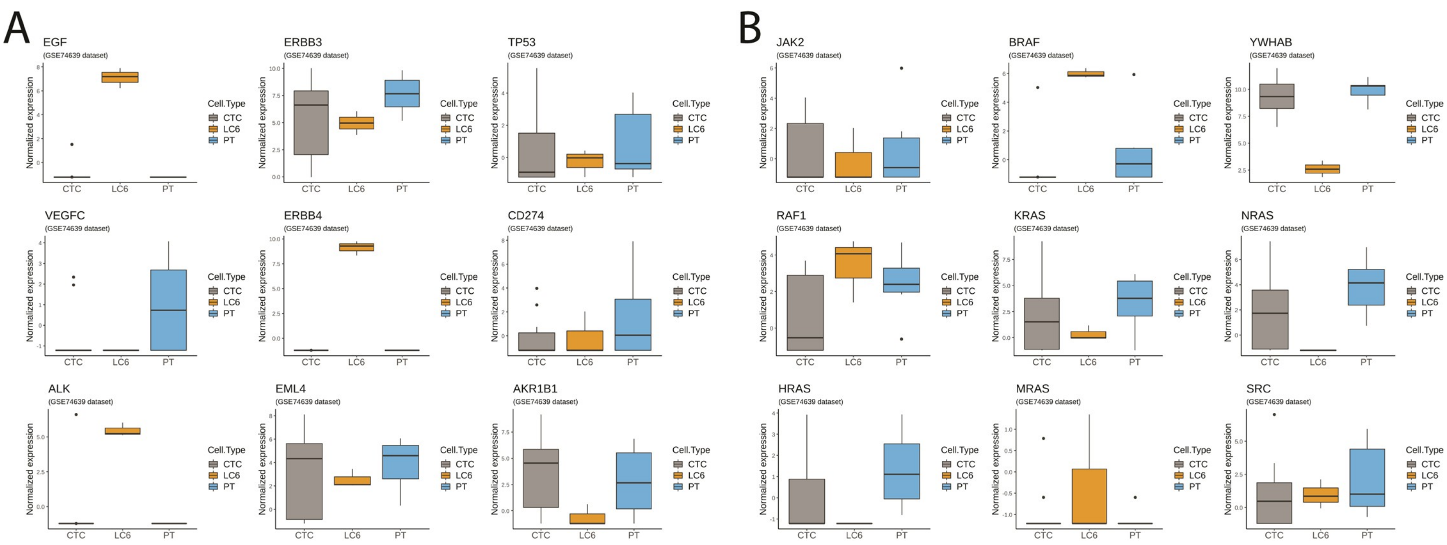

Fig 6. Sampling of potential, actionable therapeutic gene expression targets. Box plots comparing normalized gene expression levels on a logarithmic y axis from a sampling of genes among the MCCCs from patient LC6. **(A)** The selected actionable genes may be targets since existing drug interventions or new therapeutics are in development for those selected. Note the increased expression of *EGF*, *ERBB4* (*HER4*) and *ALK* compared to lung CTCs and lung primary tumor cells. **(B)** An Illustration of several genes within the *Ras/Raf* pathway. Note the relatively high expression of *BRAF* among the MCCCs suggesting a potential unseen therapeutic target not usually observed in lung primary tumor cells.

https://doi.org/10.1371/journal.pone.0306450.g006

Base (DGIdb.org), [81] we identified numerous drugs and drug candidates with high interaction scores (S3 Table in S1 File). Some examples show elevated expression in contrast to CTCs and PTs in this dataset (e.g. *ALK*, *ERBB4*, and *EGF*) while other molecular targets while clearly expressed are not noticeably upregulated. S3 Table in S1 File shows a list of corresponding therapeutics available or in development for these molecular targets. Additional research is necessary to demonstrate that inhibiting the identified targets will interfere with metastatic cluster biology.

The RNA-seq analyses from our captured MCCCs have shown the expression of several genes that are considered actionable relative to targeted therapies in the treatment of cancer. An example of a potential, previously unseen therapeutic target among the MCCCs is suggested by the elevated *ERBB4* expression as seen in Fig 6A. The ERBB4 is a tyrosine kinase receptor and a member of the epidermal growth factor receptor (EGFR) family, also known as HER4. The ERBB4 receptor is activated via ligand binding which subsequently impacts MAPKs and PI3K/AKT pathways [82]. Much research continues to elucidate the role of *ERBB4* mutations and expression among many cancer types but it may be associated with proliferative and migratory abilities of NSCLC [83]. While, *ERBB4* mutations and/or high expression levels observed among NSCLC patients have been identified, their clinical significance remains unclear [84, 85]. Elevated levels of ERBB4 among MCCCs, which can be confirmed when additional patient samples are processed, suggests a possible role in the metastatic cascade thus warranting further study.

Another gene with high expression levels within the MCCCs relative to lung CTCs and primary tumor is *BRAF* (See Fig 6B). Detection of mutated *BRAF* has been low and reported in only 2–7% of advanced NSCLC patients [86, 87]. The *BRAF* gene is part of the MAPK/ERK signaling pathway, which plays a crucial role in regulating cell growth, differentiation, and survival. Mutated *BRAF* is a well-known driver of various cancers but not usually observed with lung cancer [88]. Yet, a recent publication identified elevated wild type (WT) *BRAF* expression levels at all stages (I-IV) from lung adenocarcinoma patients and was significantly associated

with decreased overall survival [88, 89]. Identifying elevated *BRAF* expression among these rare MCCCs reinforces the need to characterize the metastatic clusters to reveal previously unseen therapeutic targets. However, the possibility that the high *BRAF* expression could have been inherited from the primary tumor and is not a unique profile of MCCCs cannot be disregarded since we do not have corresponding RNA-seq data for this patient. Furthermore, our current data is limited to expression levels and does not inform on mutational status.

An important strategy for disrupting MCCCs (aka CTC clusters) was described by Nicola Aceto's group where they identified molecules which interfered with intercellular adhesion [32]. Results from an animal model study using patient derived xenografts demonstrated an 80-fold reduction in metastatic index with the adhesion molecule inhibitor treatment group. Following this strategy, we reviewed the MCCC RNA-seq data to identify elevated expression levels for adhesion and epithelial to mesenchymal (EMT) molecules which may be associated with preparing metastatic clusters for intravasation.

Two genes associated with adhesion and EMT, *COL4A1* (Collagen, type IV, alpha 1) and *FGFR1 (*Fibroblast growth factor receptor 1), showed elevated gene expression (see Fig 7). The *COL4A1* codes for a component of type IV collagen which comprises a complex protein network involved with basement membranes supporting cell structures [90]. COL4A1 is known to promote the growth and metastasis of hepatocellular carcinoma cells [91]. Collagen, a major component of the extracellular matrix (ECM), plays a significant role in cancer metastasis. Cancer cells can alter the composition and organization of the ECM within the tumor microenvironment. Tumor cells often produce enzymes such as matrix metalloproteinases (MMPs) that degrade and remodel the ECM, allowing cancer cells to invade surrounding tissues more easily [92]. A recent publication discusses targeting specific focal adhesion and ECM receptor pathways associated with COL4A1 for liver and lung metastases. [93].

Also shown is the upregulation of *FGFR1* which has been associated with the lung cancer progression pathway in some studies [94, 95]. FGFR1 is a receptor tyrosine kinase also linked to the *MAPK/ERK* pathway and overexpression of this gene was found to promote epithelial–mesenchymal transition (EMT) plus metastasis.. Specifically, it is linked to resistance and decreased efficacy of EGFR tyrosine kinase inhibitor (TKI) treatments [94, 96].

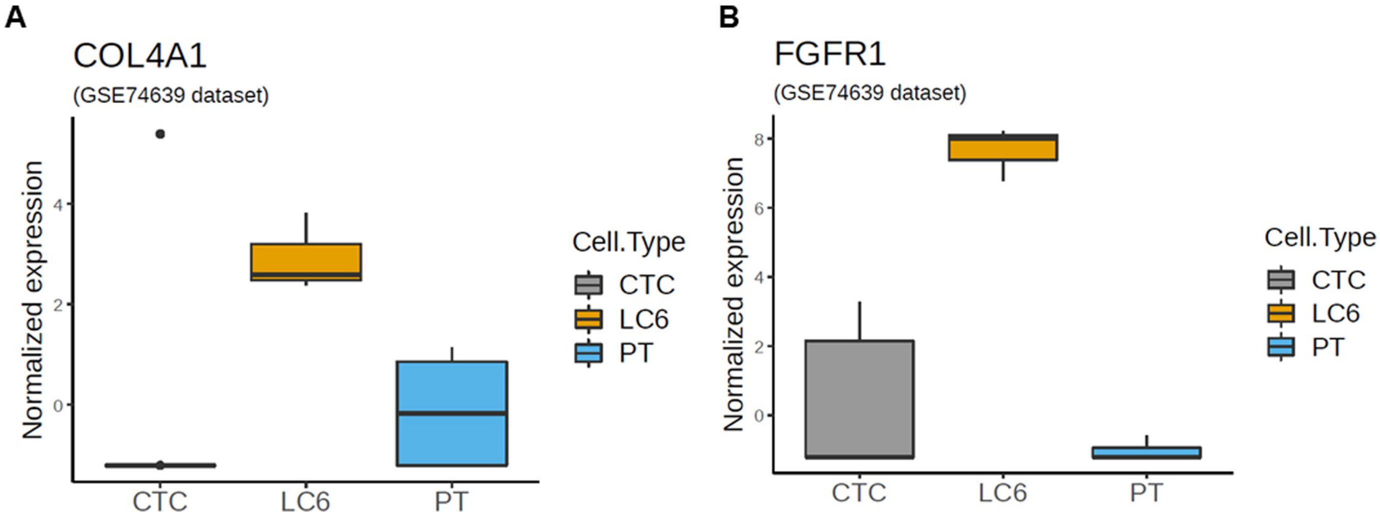

Fig 7. Gene expression correlated to intercellular adhesion and EMT. MCCCs from patient LC6 appear to show relatively high expression levels of *COL4A1* and *FGFR1* compared to individual circulating lung CTCs and lung primary tumor cells.

https://doi.org/10.1371/journal.pone.0306450.g007

Understanding the molecular signaling pathways of metastasis is essential for developing new therapeutics designed to prevent cancer's spread. Isolating and characterizing the cancer cell clusters which are more tumorigenic versus individual cancer cells is an important precursor for new anti-metastatic drug development [32, 97]. Facilitating the isolation and characterization of MCCCs will increase the number of patients analyzed for these metastatic clusters in real time, as well as during various stages of their disease. Technology that allows for frequent sampling at multiple time points may improve our understanding of the metastatic cascade during cancer's progression. Additionally, easier access to MCCCs will increase research focus on the mechanisms responsible for cancer cluster intravasation and ultimately aid the development of therapeutics to stop their genesis.

Our proof-of-concept study utilized a completely novel dual capture approach based on a highly selective, biomimetic-immunocapture 'Smart Coating' that fast tracks isolation and characterization of extremely rare MCCCs. The Smart Coating™ technology provides a positive selection bias for capturing extremely rare MCCCs directly from unprocessed whole blood.

The RNA-seq heatmap, which suggests a unique DEG profile, (Fig 5) further supports our proof-of-concept study, demonstrating the ability to capture MCCCs in circulation and characterize them to identify potential therapeutic targets. Also, identifying a unique DEG profile for MCCCs will lead to illuminating different signaling pathways that are in effect during the metastatic cascade [9, 98].

Conclusion

We acknowledge that our study has limitations. Specifically, the number of patient samples processed using fluorescently labeled mAbs to characterize the MCCCs and the corresponding RNA-seq analyses was relatively low. However, the results demonstrating that one or more MCCCs were found in all patient samples processed are highly promising. Also, captured clusters were amenable to immunofluorescent characterization and yielded high quality RNA for detailed genomic profiling. Future studies will further characterize these metastatic vectors using epigenomic and proteomic characterization. Our results show the utility of our dual capture technology to reveal previously unseen metastatic pathways. For example, identification of *ERBB4*, *BRAF*, *COL4A1* and *FGFR1* as molecular targets in highly tumorigenic MCCCs, may serve as focal points for new anti-metastatic drug development. Although the gene expression data is based on replicates from a single patient sample, our successful proof-of-concept study will enable us to expand into higher sample numbers, allowing identification of definitive expression profiles associated with NSCLC MCCCs, shared among many different patients. This will include comparisons of RNA-seq data from autologous patient samples such as lung primary tumor cells and plural effusion derived cells versus MCCCs. The simplicity and utility of our new platform has the potential to greatly expand the study of metastatic cancer cell clusters for many different solid tumor cancers.

Supporting information

S1 File.
(DOCX)

S2 File.
(PNG)

S1 Data.
(XLSX)

S2 Data.
(XLSX)

S3 Data.
(XLSX)

S4 Data.
(XLSX)

Acknowledgments

We are truly grateful for the participation of the patients that consented to provide blood samples for our study. We wish to thank Dr. Alfredo Molinolo, Director of the Biorepository and Tissue Technology Shared Resource core for Biospecimen collection at the UCSD Moores Cancer Center, for his support in obtaining the patient samples. Also, we very much appreciate the support of Dr. Sandip Patel and Dr. Greg Botta from the UCSD Moores Cancer Center. Additionally, thanks go out to Steve Head, Director of the NGS Core facility at Scripps Research Translational Institute for aiding us with the RNA-seq sample preparation and analysis. Further, we appreciate the support provided by Dr. James Evans at PhenoVista Biosciences.

Author Contributions

Conceptualization: Jeffrey K. Allen, Peter Teriete.

Formal analysis: Hector Hernandez-Vargas.

Funding acquisition: Jeffrey K. Allen, Peter Teriete.

Investigation: Kourosh Kouhmareh, Anukriti Bhadada, Francisco Downey.

Methodology: Darren Finlay.

Project administration: Jeffrey K. Allen.

Resources: Jeffrey K. Allen.

Supervision: Erika Martin.

Writing – original draft: Jeffrey K. Allen.

Writing – review & editing: Anukriti Bhadada, Jeffrey K. Allen, Peter Teriete.

References

1. Dillekås H, Rogers MS, Straume O. Are 90% of deaths from cancer caused by metastases? Cancer Medicine. 2019; 8(12):5574–6. https://doi.org/10.1002/cam4.2474 PMID: 31397113
2. Tamura T, Kurishima K, Nakazawa K, Kagohashi K, Ishikawa H, Satoh H, et al. Specific organ metastases and survival in metastatic non-small-cell lung cancer. Molecular and Clinical Oncology. 2015; 3 (1):217–21. https://doi.org/10.3892/mco.2014.410 PMID: 25469298
3. Disibio G, French SW. Metastatic patterns of cancers: results from a large autopsy study. Arch Pathol Lab Med. 2008; 132(6):931–9. https://doi.org/10.5858/2008-132-931-MPOCRF PMID: 18517275
4. Riihimaki M, Hemminki A, Fallah M, Thomsen H, Sundquist K, Sundquist J, et al. Metastatic sites and survival in lung cancer. Lung Cancer. 2014; 86(1):78–84. https://doi.org/10.1016/j.lungcan.2014.07.020 PMID: 25130083
5. Thallinger C, Füreder T, Preusser M, Heller G, Müllauer L, Höller C, et al. Review of cancer treatment with immune checkpoint inhibitors: Current concepts, expectations, limitations and pitfalls. Wien Klin Wochenschr. 2018; 130(3–4):85–91. https://doi.org/10.1007/s00508-017-1285-9 PMID: 29098404

6. Ganesh K, Massagué J. Targeting metastatic cancer. Nat Med. 2021; 27(1):34–44. https://doi.org/10.1038/s41591-020-01195-4 PMID: 33442008
7. Caswell DR, Swanton C. The role of tumour heterogeneity and clonal cooperativity in metastasis, immune evasion and clinical outcome. BMC Med. 2017; 15(1):133. https://doi.org/10.1186/s12916-017-0900-y PMID: 28716075
8. Anderson RL, Balasas T, Callaghan J, Coombes RC, Evans J, Hall JA, et al. A framework for the development of effective anti-metastatic agents. Nat Rev Clin Oncol. 2019; 16(3):185–204. https://doi.org/10.1038/s41571-018-0134-8 PMID: 30514977
9. Riggi N, Aguet M, Stamenkovic I. Cancer Metastasis: A Reappraisal of Its Underlying Mechanisms and Their Relevance to Treatment. Annu Rev Pathol. 2018; 13:117–40. https://doi.org/10.1146/annurev-pathol-020117-044127 PMID: 29068753
10. Wang WC, Zhang XF, Peng J, Li XF, Wang AL, Bie YQ, et al. Survival Mechanisms and Influence Factors of Circulating Tumor Cells. Biomed Res Int. 2018; 2018:6304701. https://doi.org/10.1155/2018/6304701 PMID: 30515411
11. Aceto N, Bardia A, Miyamoto DT, Donaldson MC, Wittner BS, Spencer JA, et al. Circulating tumor cell clusters are oligoclonal precursors of breast cancer metastasis. Cell. 2014; 158(5):1110–22. https://doi.org/10.1016/j.cell.2014.07.013 PMID: 25171411
12. Cheung KJ, Padmanaban V, Silvestri V, Schipper K, Cohen JD, Fairchild AN, et al. Polyclonal breast cancer metastases arise from collective dissemination of keratin 14-expressing tumor cell clusters. Proc Natl Acad Sci U S A. 2016; 113(7):E854–63. https://doi.org/10.1073/pnas.1508541113 PMID: 26831077
13. Wrenn ED, Yamamoto A, Moore BM, Huang Y, McBirney M, Thomas AJ, et al. Regulation of Collective Metastasis by Nanolumenal Signaling. Cell. 2020; 183(2):395–410.e19. https://doi.org/10.1016/j.cell.2020.08.045 PMID: 33007268
14. Schuster E, Taftaf R, Reduzzi C, Albert MK, Romero-Calvo I, Liu H. Better together: circulating tumor cell clustering in metastatic cancer. Trends Cancer. 2021; 7(11):1020–32. https://doi.org/10.1016/j.trecan.2021.07.001 PMID: 34481763
15. Wan S, Kim TH, Smith KJ, Delaney R, Park GS, Guo H, et al. New Labyrinth Microfluidic Device Detects Circulating Tumor Cells Expressing Cancer Stem Cell Marker and Circulating Tumor Microemboli in Hepatocellular Carcinoma. Sci Rep. 2019; 9(1):18575. https://doi.org/10.1038/s41598-019-54960-y PMID: 31819089
16. Liu X, Adorno-Cruz V, Chang YF, Jia Y, Kawaguchi M, Dashzeveg NK, et al. EGFR inhibition blocks cancer stem cell clustering and lung metastasis of triple negative breast cancer. Theranostics. 2021; 11(13):6632–43. https://doi.org/10.7150/thno.57706 PMID: 33995681
17. Xu K, Wang R, Xie H, Hu L, Wang C, Xu J, et al. Single-cell RNA sequencing reveals cell heterogeneity and transcriptome profile of breast cancer lymph node metastasis. Oncogenesis. 2021; 10(10):66. https://doi.org/10.1038/s41389-021-00355-6 PMID: 34611125
18. Marchant CL, Malmi-Kakkada AN, Espina JA, Barriga EH. Cell clusters softening triggers collective cell migration in vivo. Nat Mater. 2022; 21(11):1314–23. https://doi.org/10.1038/s41563-022-01323-0 PMID: 35970965
19. Ring A, Nguyen-Sträuli BD, Wicki A, Aceto N. Biology, vulnerabilities and clinical applications of circulating tumour cells. Nat Rev Cancer. 2023; 23(2):95–111. https://doi.org/10.1038/s41568-022-00536-4 PMID: 36494603
20. Piñeiro R, Martínez-Pena I, López-López R. Relevance of CTC Clusters in Breast Cancer Metastasis. Adv Exp Med Biol. 2020; 1220:93–115. https://doi.org/10.1007/978-3-030-35805-1_7 PMID: 32304082
21. Castro-Giner F, Aceto N. Tracking cancer progression: from circulating tumor cells to metastasis. Genome Med. 2020; 12(1):31. https://doi.org/10.1186/s13073-020-00728-3 PMID: 32192534
22. Herath S, Razavi Bazaz S, Monkman J, Ebrahimi Warkiani M, Richard D, O'Byrne K, et al. Circulating tumor cell clusters: Insights into tumour dissemination and metastasis. Expert Rev Mol Diagn. 2020; 20(11):1139–47. https://doi.org/10.1080/14737159.2020.1846523 PMID: 33140979
23. Arnoletti JP, Fanaian N, Reza J, Sause R, Almodovar AJ, Srivastava M, et al. Pancreatic and bile duct cancer circulating tumor cells (CTC) form immune-resistant multi-cell type clusters in the portal venous circulation. Cancer Biol Ther. 2018; 19(10):887–97. https://doi.org/10.1080/15384047.2018.1480292 PMID: 30067440
24. Jia D, Li X, Bocci F, Tripathi S, Deng Y, Jolly MK, et al. Quantifying Cancer Epithelial-Mesenchymal Plasticity and its Association with Stemness and Immune Response. J Clin Med. 2019; 8(5). https://doi.org/10.3390/jcm8050725 PMID: 31121840

25. Uchôa Guimarães CT, Ferreira Martins NN, Cristina da Silva Oliveira K, Almeida CM, Pinheiro TM, Gigek CO, et al. Liquid biopsy provides new insights into gastric cancer. Oncotarget. 2018; 9 (19):15144–56. https://doi.org/10.18632/oncotarget.24540 PMID: 29599934

26. Zajac O, Raingeaud J, Libanje F, Lefebvre C, Sabino D, Martins I, et al. Tumour spheres with inverted polarity drive the formation of peritoneal metastases in patients with hypermethylated colorectal carcinomas. Nat Cell Biol. 2018; 20(3):296–306. https://doi.org/10.1038/s41556-017-0027-6 PMID: 29403038

27. Sharma U, Medina-Saenz K, Miller PC, Troness B, Spartz A, Sandoval-Leon A, et al. Heterotypic clustering of circulating tumor cells and circulating cancer-associated fibroblasts facilitates breast cancer metastasis. Breast Cancer Res Treat. 2021; 189(1):63–80. https://doi.org/10.1007/s10549-021-06299-0 PMID: 34216317

28. Szczerba BM, Castro-Giner F, Vetter M, Krol I, Gkountela S, Landin J, et al. Neutrophils escort circulating tumour cells to enable cell cycle progression. Nature. 2019; 566(7745):553–7. https://doi.org/10.1038/s41586-019-0915-y PMID: 30728496

29. Heeke S, Mograbi B, Alix-Panabières C, Hofman P. Never Travel Alone: The Crosstalk of Circulating Tumor Cells and the Blood Microenvironment. Cells. 2019; 8(7). https://doi.org/10.3390/cells8070714 PMID: 31337010

30. Nakamura K, Smyth MJ. Immunoediting of cancer metastasis by NK cells. Nat Cancer. 2020; 1(7):670–1. https://doi.org/10.1038/s43018-020-0081-z PMID: 35122037

31. Chan IS, Knútsdóttir H, Ramakrishnan G, Padmanaban V, Warrier M, Ramirez JC, et al. Cancer cells educate natural killer cells to a metastasis-promoting cell state. J Cell Biol. 2020; 219(9). https://doi.org/10.1083/jcb.202001134 PMID: 32645139

32. Gkountela S, Castro-Giner F, Szczerba BM, Vetter M, Landin J, Scherrer R, et al. Circulating Tumor Cell Clustering Shapes DNA Methylation to Enable Metastasis Seeding. Cell. 2019; 176(1–2):98–112. e14. https://doi.org/10.1016/j.cell.2018.11.046 PMID: 30633912

33. Sharma S, Zhuang R, Long M, Pavlovic M, Kang Y, Ilyas A, et al. Circulating tumor cell isolation, culture, and downstream molecular analysis. Biotechnol Adv. 2018; 36(4):1063–78. https://doi.org/10.1016/j.biotechadv.2018.03.007 PMID: 29559380

34. Pugia M, Magbanua MJM, Park JW. Enrichment and Detection of Circulating Tumor Cells and Other Rare Cell Populations by Microfluidic Filtration. Adv Exp Med Biol. 2017; 994:119–31. https://doi.org/10.1007/978-3-319-55947-6_6 PMID: 28560671

35. Kojić N, Milošević M, Petrović D, Isailović V, Sarioglu AF, Haber DA, et al. A computational study of circulating large tumor cells traversing microvessels. Comput Biol Med. 2015; 63:187–95. https://doi.org/10.1016/j.compbiomed.2015.05.024 PMID: 26093786

36. Takagi H, Dong L, Kuczler MD, Lombardo K, Hirai M, Amend SR, et al. Analysis of the Circulating Tumor Cell Capture Ability of a Slit Filter-Based Method in Comparison to a Selection-Free Method in Multiple Cancer Types. Int J Mol Sci. 2020; 21(23). https://doi.org/10.3390/ijms21239031 PMID: 33261132

37. Bergman R, Afifi A, Heidger P. Atlas of Microscopic Anatomy. Michael P. D'Alessandro MD, editor: WB Saunders; 1996.

38. Yamamoto A, Doak AE, Cheung KJ. Orchestration of Collective Migration and Metastasis by Tumor Cell Clusters. Annu Rev Pathol. 2023; 18:231–56. https://doi.org/10.1146/annurev-pathmechdis-031521-023557 PMID: 36207009

39. Amintas S, Bedel A, Moreau-Gaudry F, Boutin J, Buscail L, Merlio JP, et al. Circulating Tumor Cell Clusters: United We Stand Divided We Fall. Int J Mol Sci. 2020; 21(7). https://doi.org/10.3390/ijms21072653 PMID: 32290245

40. Fedosov DA, Gompper G. White blood cell margination in microcirculation. Soft Matter. 2014; 10 (17):2961–70. https://doi.org/10.1039/c3sm52860j PMID: 24695813

41. Wang S, Ye T, Li G, Zhang X, Shi H. Margination and adhesion dynamics of tumor cells in a real microvascular network. PLoS Comput Biol. 2021; 17(2):e1008746. https://doi.org/10.1371/journal.pcbi.1008746 PMID: 33606686

42. Teriete P, Banerji S, Noble M, Blundell CD, Wright AJ, Pickford AR, et al. Structure of the regulatory hyaluronan binding domain in the inflammatory leukocyte homing receptor CD44. Mol Cell. 2004; 13 (4):483–96. https://doi.org/10.1016/s1097-2765(04)00080-2 PMID: 14992719

43. Liu X, Taftaf R, Kawaguchi M, Chang YF, Chen W, Entenberg D, et al. Homophilic CD44 Interactions Mediate Tumor Cell Aggregation and Polyclonal Metastasis in Patient-Derived Breast Cancer Models. Cancer Discov. 2019; 9(1):96–113. https://doi.org/10.1158/2159-8290.CD-18-0065 PMID: 30361447

44. Kawaguchi M, Dashzeveg N, Cao Y, Jia Y, Liu X, Shen Y, et al. Extracellular Domains I and II of cell-surface glycoprotein CD44 mediate its trans-homophilic dimerization and tumor cluster aggregation. J Biol Chem. 2020; 295(9):2640–9. https://doi.org/10.1074/jbc.RA119.010252 PMID: 31969394

45. San Juan BP, Garcia-Leon MJ, Rangel L, Goetz JG, Chaffer CL. The Complexities of Metastasis. Cancers (Basel). 2019; 11(10). https://doi.org/10.3390/cancers11101575 PMID: 31623163

46. Kapeleris J, Zou H, Qi Y, Gu Y, Li J, Schoning J, et al. Cancer stemness contributes to cluster formation of colon cancer cells and high metastatic potentials. Clin Exp Pharmacol Physiol. 2020; 47(5):838–47. https://doi.org/10.1111/1440-1681.13247 PMID: 31883392

47. Rodrigues P, Vanharanta S. Circulating Tumor Cells: Come Together, Right Now, Over Metastasis. Cancer Discov. 2019; 9(1):22–4. https://doi.org/10.1158/2159-8290.CD-18-1285 PMID: 30626605

48. Shaner SW, Allen JK, Felderman M, Pasko ET, Wimer CD, Cosford NDP, et al. Design and production of a novel microfluidic device for the capture and isolation of circulating tumor cell clusters. AIP Advances. 2019; 9(6):065313.

49. Lee KY, Mooney DJ. Alginate: properties and biomedical applications. Prog Polym Sci. 2012; 37 (1):106–26. https://doi.org/10.1016/j.progpolymsci.2011.06.003 PMID: 22125349

50. Giannopoulou E, Nikolakopoulos A, Kotsirilou D, Lampropoulou A, Raftopoulou S, Papadimitriou E, et al. Epidermal growth factor receptor status and Notch inhibition in non-small cell lung cancer cells. J Biomed Sci. 2015; 22:98. https://doi.org/10.1186/s12929-015-0196-1 PMID: 26497899

51. Salgia R. MET in Lung Cancer: Biomarker Selection Based on Scientific Rationale. Mol Cancer Ther. 2017; 16(4):555–65. https://doi.org/10.1158/1535-7163.MCT-16-0472 PMID: 28373408

52. Scharpenseel H, Hanssen A, Loges S, Mohme M, Bernreuther C, Peine S, et al. EGFR and HER3 expression in circulating tumor cells and tumor tissue from non-small cell lung cancer patients. Sci Rep. 2019; 9(1):7406. https://doi.org/10.1038/s41598-019-43678-6 PMID: 31092882

53. Wong KHK, Tessier SN, Miyamoto DT, Miller KL, Bookstaver LD, Carey TR, et al. Whole blood stabilization for the microfluidic isolation and molecular characterization of circulating tumor cells. Nat Commun. 2017; 8(1):1733. https://doi.org/10.1038/s41467-017-01705-y PMID: 29170510

54. Andree KC, Mentink A, Zeune LL, Terstappen L, Stoecklein NH, Neves RP, et al. Toward a real liquid biopsy in metastatic breast and prostate cancer: Diagnostic LeukApheresis increases CTC yields in a European prospective multicenter study (CTCTrap). Int J Cancer. 2018; 143(10):2584–91. https://doi.org/10.1002/ijc.31752 PMID: 30006930

55. Joosse SA, Gorges TM, Pantel K. Biology, detection, and clinical implications of circulating tumor cells. EMBO Mol Med. 2015; 7(1):1–11. https://doi.org/10.15252/emmm.201303698 PMID: 25398926

56. Robinson MD, Oshlack A. A scaling normalization method for differential expression analysis of RNA-seq data. Genome Biol. 2010; 11(3):R25. https://doi.org/10.1186/gb-2010-11-3-r25 PMID: 20196867

57. Zheng Y, Miyamoto DT, Wittner BS, Sullivan JP, Aceto N, Jordan NV, et al. Expression of β-globin by cancer cells promotes cell survival during blood-borne dissemination. Nat Commun. 2017; 8:14344.

58. Wittner BS. GSE74639: RNA-Seq of Single Lung Circulating Tumor Cells (CTCs) and primary Tumor Cells from an orthotopic lung xenograft model: Massachusetts General Hospital, Center for Cancer Research; 2015 [Available from: https://www.ncbi.nlm.nih.gov/geo/query/acc.cgi?acc=GSE74639].

59. Walker JR. **Sample GSM18949**: Genomics Institute of the Novartis Research Foundation; Mar 19, 2004 [Available from: https://www.ncbi.nlm.nih.gov/geo/query/acc.cgi?acc=GSM18949].

60. Walker JR. **Sample GSM18950**: Genomics Institute of the Novartis Research Foundation; Mar 19, 2004 [cited 2023 Mar 3, 2023]. Available from: https://www.ncbi.nlm.nih.gov/geo/query/acc.cgi?acc=GSM18950.

61. Newton K, Dixit VM. Signaling in innate immunity and inflammation. Cold Spring Harb Perspect Biol. 2012; 4(3). https://doi.org/10.1101/cshperspect.a006049 PMID: 22296764

62. Zarbock A, Ley K, McEver RP, Hidalgo A. Leukocyte ligands for endothelial selectins: specialized glycoconjugates that mediate rolling and signaling under flow. Blood. 2011; 118(26):6743–51. https://doi.org/10.1182/blood-2011-07-343566 PMID: 22021370

63. Yagi S, Koh Y, Akamatsu H, Kanai K, Hayata A, Tokudome N, et al. Development of an automated size-based filtration system for isolation of circulating tumor cells in lung cancer patients. PLoS One. 2017; 12(6):e0179744. https://doi.org/10.1371/journal.pone.0179744 PMID: 28640869

64. Christophis C, Taubert I, Meseck GR, Schubert M, Grunze M, Ho AD, et al. Shear stress regulates adhesion and rolling of CD44+ leukemic and hematopoietic progenitor cells on hyaluronan. Biophys J. 2011; 101(3):585–93. https://doi.org/10.1016/j.bpj.2011.05.045 PMID: 21806926

65. Hümmer J, Koc J, Rosenhahn A, Lee-Thedieck C. Microfluidic Shear Force Assay to Determine Cell Adhesion Forces. Methods Mol Biol. 2019; 2017:71–84. https://doi.org/10.1007/978-1-4939-9574-5_6 PMID: 31197769

66. Bethune G, Bethune D, Ridgway N, Xu Z. Epidermal growth factor receptor (EGFR) in lung cancer: an overview and update. J Thorac Dis. 2010; 2(1):48–51. PMID: 22263017
67. Au SH, Storey BD, Moore JC, Tang Q, Chen YL, Javaid S, et al. Clusters of circulating tumor cells traverse capillary-sized vessels. Proc Natl Acad Sci U S A. 2016; 113(18):4947–52. https://doi.org/10.1073/pnas.1524448113 PMID: 27091969
68. Hao SJ, Wan Y, Xia YQ, Zou X, Zheng SY. Size-based separation methods of circulating tumor cells. Adv Drug Deliv Rev. 2018; 125:3–20. https://doi.org/10.1016/j.addr.2018.01.002 PMID: 29326054
69. Lim M, Park S, Jeong HO, Park SH, Kumar S, Jang A, et al. Circulating Tumor Cell Clusters Are Cloaked with Platelets and Correlate with Poor Prognosis in Unresectable Pancreatic Cancer. Cancers (Basel). 2021; 13(21). https://doi.org/10.3390/cancers13215272 PMID: 34771436
70. Anvari S, Osei E, Maftoon N. Interactions of platelets with circulating tumor cells contribute to cancer metastasis. Sci Rep. 2021; 11(1):15477. https://doi.org/10.1038/s41598-021-94735-y PMID: 34326373
71. Johnson P, Ruffell B. CD44 and its role in inflammation and inflammatory diseases. Inflamm Allergy Drug Targets. 2009; 8(3):208–20. https://doi.org/10.2174/187152809788680994 PMID: 19601881
72. Chen Q, Zou J, He Y, Pan Y, Yang G, Zhao H, et al. A narrative review of circulating tumor cells clusters: A key morphology of cancer cells in circulation promote hematogenous metastasis. Front Oncol. 2022; 12:944487. https://doi.org/10.3389/fonc.2022.944487 PMID: 36059616
73. Fridrichova I, Kalinkova L, Ciernikova S. Clinical Relevancy of Circulating Tumor Cells in Breast Cancer: Epithelial or Mesenchymal Characteristics, Single Cells or Clusters? Int J Mol Sci. 2022; 23(20). https://doi.org/10.3390/ijms232012141 PMID: 36292996
74. Al Barashdi MA, Ali A, McMullin MF, Mills K. Protein tyrosine phosphatase receptor type C (PTPRC or CD45). J Clin Pathol. 2021; 74(9):548–52. https://doi.org/10.1136/jclinpath-2020-206927 PMID: 34039664
75. Skytthe MK, Graversen JH, Moestrup SK. Targeting of CD163(+) Macrophages in Inflammatory and Malignant Diseases. Int J Mol Sci. 2020; 21(15). https://doi.org/10.3390/ijms21155497 PMID: 32752088
76. Dufour EM, Deroche A, Bae Y, Muller WA. CD99 is essential for leukocyte diapedesis in vivo. Cell Commun Adhes. 2008; 15(4):351–63. https://doi.org/10.1080/15419060802442191 PMID: 18923973
77. Song Y, Ahn J, Suh Y, Davis ME, Lee K. Identification of novel tissue-specific genes by analysis of microarray databases: a human and mouse model. PLoS One. 2013; 8(5):e64483. https://doi.org/10.1371/journal.pone.0064483 PMID: 23741331
78. Walker JR. **Sample GSM18950**: Genomics Institute of the Novartis Research Foundation; [cited 2023 Mar 3, 2023]. Available from: https://www.ncbi.nlm.nih.gov/geo/query/acc.cgi?acc=GSM18950.
79. Zheng Y. GSE74639: RNA-Seq of Single Lung Circulating Tumor Cells (CTCs) and primary Tumor Cells from an orthotopic lung xenograft model: Massachusetts General Hospital Center For Cancer Research; Mar 2, 2017 [Available from: https://www.ncbi.nlm.nih.gov/geo/query/acc.cgi?acc=GSE74639.
80. Moisés J, Navarro A, Santasusagna S, Viñolas N, Molins L, Ramirez J, et al. NKX2-1 expression as a prognostic marker in early-stage non-small-cell lung cancer. BMC Pulm Med. 2017; 17(1):197. https://doi.org/10.1186/s12890-017-0542-z PMID: 29237428
81. Freshour SL, Kiwala S, Cotto KC, Coffman AC, McMichael JF, Song JJ, et al. Integration of the Drug-Gene Interaction Database (DGIdb 4.0) with open crowdsource efforts. Nucleic Acids Res. 2021; 49 (D1):D1144–d51. https://doi.org/10.1093/nar/gkaa1084 PMID: 33237278
82. Wang Z. ErbB Receptors and Cancer. Methods Mol Biol. 2017; 1652:3–35. https://doi.org/10.1007/978-1-4939-7219-7_1 PMID: 28791631
83. El-Gamal MI, Mewafi NH, Abdelmotteleb NE, Emara MA, Tarazi H, Sbenati RM, et al. A Review of HER4 (ErbB4) Kinase, Its Impact on Cancer, and Its Inhibitors. Molecules. 2021; 26(23).
84. Tomizawa K, Suda K, Onozato R, Kuwano H, Yatabe Y, Mitsudomi T. Analysis of ERBB4 mutations and expression in japanese patients with lung cancer. J Thorac Oncol. 2010; 5(11):1859–61. https://doi.org/10.1097/JTO.0b013e3181f1c433 PMID: 20975381
85. Kurppa KJ, Denessiouk K, Johnson MS, Elenius K. Activating ERBB4 mutations in non-small cell lung cancer. Oncogene. 2016; 35(10):1283–91. https://doi.org/10.1038/onc.2015.185 PMID: 26050618
86. Skoulidis F, Heymach JV. Co-occurring genomic alterations in non-small-cell lung cancer biology and therapy. Nat Rev Cancer. 2019; 19(9):495–509.
87. Tissot C, Couraud S, Tanguy R, Bringuier PP, Girard N, Souquet PJ. Clinical characteristics and outcome of patients with lung cancer harboring BRAF mutations. Lung Cancer. 2016; 91:23–8. https://doi.org/10.1016/j.lungcan.2015.11.006 PMID: 26711930

88. Sumimoto H, Imabayashi F, Iwata T, Kawakami Y. The BRAF-MAPK signaling pathway is essential for cancer-immune evasion in human melanoma cells. J Exp Med. 2006; 203(7):1651–6. https://doi.org/10.1084/jem.20051848 PMID: 16801397

89. Dora D, Vörös I, Varga ZV, Takacs P, Teglasi V, Moldvay J, et al. BRAF RNA is prognostic and widely expressed in lung adenocarcinoma. Transl Lung Cancer Res. 2023; 12(1):27–41. https://doi.org/10.21037/tlcr-22-449 PMID: 36762067

90. Zeng X, Wang HY, Wang YP, Bai SY, Pu K, Zheng Y, et al. COL4A family: potential prognostic biomarkers and therapeutic targets for gastric cancer. Transl Cancer Res. 2020; 9(9):5218–32. https://doi.org/10.21037/tcr-20-517 PMID: 35117889

91. Wang T, Jin H, Hu J, Li X, Ruan H, Xu H, et al. COL4A1 promotes the growth and metastasis of hepatocellular carcinoma cells by activating FAK-Src signaling. J Exp Clin Cancer Res. 2020; 39(1):148. https://doi.org/10.1186/s13046-020-01650-7 PMID: 32746865

92. Kleiner DE, Stetler-Stevenson WG. Matrix metalloproteinases and metastasis. Cancer Chemother Pharmacol. 1999; 43 Suppl:S42-51. https://doi.org/10.1007/s002800051097 PMID: 10357558

93. DaSilva NA, Barlock BJ, Guha P, Ghosh CC, Trebino CE, Camberg JL, et al. Proteomic signatures of myeloid derived suppressor cells from liver and lung metastases reveal functional divergence and potential therapeutic targets. Cell Death Discov. 2021; 7(1):232. https://doi.org/10.1038/s41420-021-00621-x PMID: 34482371

94. Vad-Nielsen J, Gammelgaard KR, Daugaard TF, Nielsen AL. Cause-and-Effect relationship between FGFR1 expression and epithelial-mesenchymal transition in EGFR-mutated non-small cell lung cancer cells. Lung Cancer. 2019; 132:132–40. https://doi.org/10.1016/j.lungcan.2019.04.023 PMID: 31097086

95. Wang K, Ji W, Yu Y, Li Z, Niu X, Xia W, et al. FGFR1-ERK1/2-SOX2 axis promotes cell proliferation, epithelial-mesenchymal transition, and metastasis in FGFR1-amplified lung cancer. Oncogene. 2018; 37(39):5340–54. https://doi.org/10.1038/s41388-018-0311-3 PMID: 29858603

96. Gammelgaard KR, Vad-Nielsen J, Clement MS, Weiss S, Daugaard TF, Dagnæs-Hansen F, et al. Up-Regulated FGFR1 Expression as a Mediator of Intrinsic TKI Resistance in EGFR-Mutated NSCLC. Transl Oncol. 2019; 12(3):432–40. https://doi.org/10.1016/j.tranon.2018.11.017 PMID: 30562682

97. Scheidmann MC, Castro-Giner F, Strittmatter K, Krol I, Paasinen-Sohns A, Scherrer R, et al. An In Vivo CRISPR Screen Identifies Stepwise Genetic Dependencies of Metastatic Progression. Cancer Res. 2022; 82(4):681–94. https://doi.org/10.1158/0008-5472.CAN-21-3908 PMID: 34916221

98. Fabisiewicz A, Grzybowska E. CTC clusters in cancer progression and metastasis. Med Oncol. 2017; 34(1):12. https://doi.org/10.1007/s12032-016-0875-0 PMID: 28012133
